# The right ventricle in tetralogy of Fallot: adaptation to sequential loading

**DOI:** 10.3389/fped.2023.1098248

**Published:** 2023-03-16

**Authors:** Rahi S. Alipour Symakani, Wouter J. van Genuchten, Lotte M. Zandbergen, Surya Henry, Yannick J. H. J. Taverne, Daphne Merkus, Willem A. Helbing, Beatrijs Bartelds

**Affiliations:** ^1^ Department of Pediatrics, Division of Pediatric Cardiology, Erasmus Medical Center, Sophia Children’s Hospital, Rotterdam, Netherlands; ^2^ Department of Cardiology, Division of Experimental Cardiology, Erasmus Medical Center, Rotterdam, Netherlands; ^3^ Department of Cardiothoracic Surgery, Erasmus Medical Center, Rotterdam, Netherlands; ^4^ Walter Brendel Center of Experimental Medicine (WBex), University Clinic Munich, Munich, Germany; ^5^ Department of Cell Biology, Erasmus Medical Center, Rotterdam, Netherlands; ^6^ German Center for Cardiovascular Research (DZHK), Partner Site Munich, Munich Heart Alliance (MHA), Munich, Germany

**Keywords:** tetralogy of Fallot, right ventricular dysfunction (RV dysfunction), myocardial adaptation, pulmonary regurgitation, congenital heart disease, ventricular hypertrophy, animal models

## Abstract

Right ventricular dysfunction is a major determinant of outcome in patients with complex congenital heart disease, as in tetralogy of Fallot. In these patients, right ventricular dysfunction emerges after initial pressure overload and hypoxemia, which is followed by chronic volume overload due to pulmonary regurgitation after corrective surgery. Myocardial adaptation and the transition to right ventricular failure remain poorly understood. Combining insights from clinical and experimental physiology and myocardial (tissue) data has identified a disease phenotype with important distinctions from other types of heart failure. This phenotype of the right ventricle in tetralogy of Fallot can be described as a syndrome of dysfunctional characteristics affecting both contraction and filling. These characteristics are the end result of several adaptation pathways of the cardiomyocytes, myocardial vasculature and extracellular matrix. As long as the long-term outcome of surgical correction of tetralogy of Fallot remains suboptimal, other treatment strategies need to be explored. Novel insights in failure of adaptation and the role of cardiomyocyte proliferation might provide targets for treatment of the (dysfunctional) right ventricle under stress.

## 1. Introduction

Tetralogy of Fallot (TOF) is the most prevalent diagnosis in most follow up cohorts of complex congenital heart disease (CHD) (1, 2). Present day practice of direct early surgical correction significantly reduced mortality and the average life expectancy has risen to well above 60 years of age (3, 4). Unfortunately, the growing cohort of survivors with TOF exhibits a number of late sequelae, e.g., development of heart failure, necessity of re-interventions and arrhythmogenic vulnerability (5).

Heart failure is the most common cause of death in survivors with CHD and its prevention in TOF is one the three major focus areas in research (3, 6). Heart failure in TOF is mostly right ventricular (RV) failure and in many cases associated with chronic pulmonary regurgitation (PR), a common complication following the surgical intervention aimed to relieve pulmonary stenosis (7). The development of the RV failure has been studied extensively in the last decades, albeit mostly in models of acquired RV pressure overload and pulmonary hypertension, a disease phenotype with several major dissimilarities from TOF and chronic PR.

Currently, medical treatment options for RV failure are lacking (8, 9). The most common treatment for patients with PR is pulmonary valve replacement. Timing of pulmonary valve replacement is highly debated, as repeat interventions due to child growth and valve graft degeneration are major limiting factors in its application. Furthermore, re-intervention for pulmonary valve replacement also fails to reduce incidence of RV failure or death, despite improvement of RV volumes and symptoms (10, 11).

Identification of novel targets for treatment of RV failure is necessary, which requires a deeper understanding of the shift from adaptive remodeling to failure. To be able to develop strategies to predict, detect, treat, or prevent heart failure and other late sequelae in TOF, a thorough understanding of the myriad of drivers of disease progression is needed. The drivers of other late sequelae, e.g., malignant arrhythmia, might overlap with those of RV dysfunction but will not be specifically discussed.

This narrative review will focus on what is known of the mechanisms of RV dysfunction in TOF, chronic PR and present knowledge-gaps. We aim to integrate information from functional, cellular and molecular studies with clinical data and explore potential targets for therapy.

## 2. Sequential loading: abnormal loading during growth and development

**Figure 1:**
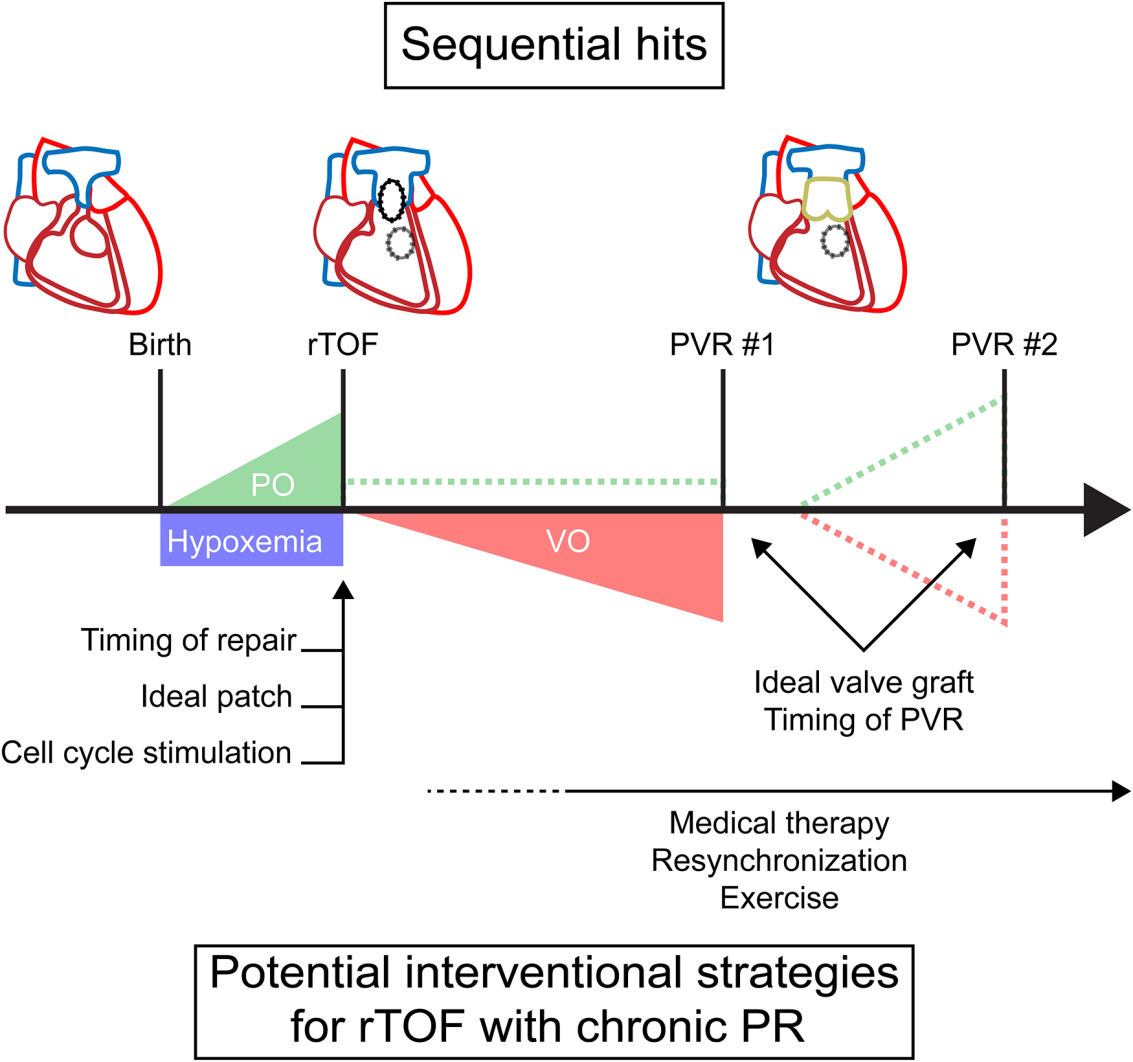
A timeline of sequential loading in tetralogy of Fallot with timing of potential interventions. Dashed lines represent potential residual or recurrent overload. PO, pressure overload; PR, pulmonary regurgitation; PVR, pulmonary valve replacement; rTOF, repaired tetralogy of Fallot; VO, volume overload.

Patients with TOF experience a unique sequence of abnormal loading conditions, which affects myocardial adaptation and ultimately cardiac function. Unlike in the normal heart, there is no unloading of the RV at birth when separation of the systemic and pulmonary circulation occurs. Due to right ventricular outflow tract (RVOT) obstruction, the postnatal RV remains subjected to pressure overload*,* as in the fetal circulation, and the myocardium retains some of its fetal characteristics after birth (12). In addition, most infants with unrepaired TOF develop hypoxemia due to ventricular right-left shunting. This combination of pressure overload and hypoxemia persists and requires corrective surgery to prevent imminent RV failure (13). After corrective surgery, repaired TOF (rTOF) patients experience different degrees of PR inducing volume overload (Figure 1). This volume overload is introduced in an already pressure overloaded RV and will affect RV function differently opposed to introducing isolated PR in an otherwise healthy RV (14). RVOT structure and function may be altered due to patch repair and right bundle branch block may eventually predispose to dyssynchrony. In some cases, residual RVOT obstruction remains present after corrective surgery. Unloading of volume and/or pressure overload can occur after pulmonary valve replacement. For isolated pressure overload, other invasive treatment options may be required. Limited valve graft durability, however, often results in recurrence of PR, RVOT obstruction or a combination of both (15). This might require repetitive pulmonary valve replacements, posing a heavy burden on patients without the guarantee of freedom from adverse events (10, 11).

Thus, this pattern of sequential loading and hypoxemia gives rise to a complex physiology. In understanding this response, the separate mechanisms, their interactions and the time-relationship should be taken into account.

## 3. The phenotype of right ventricular dysfunction

**Figure 2:**
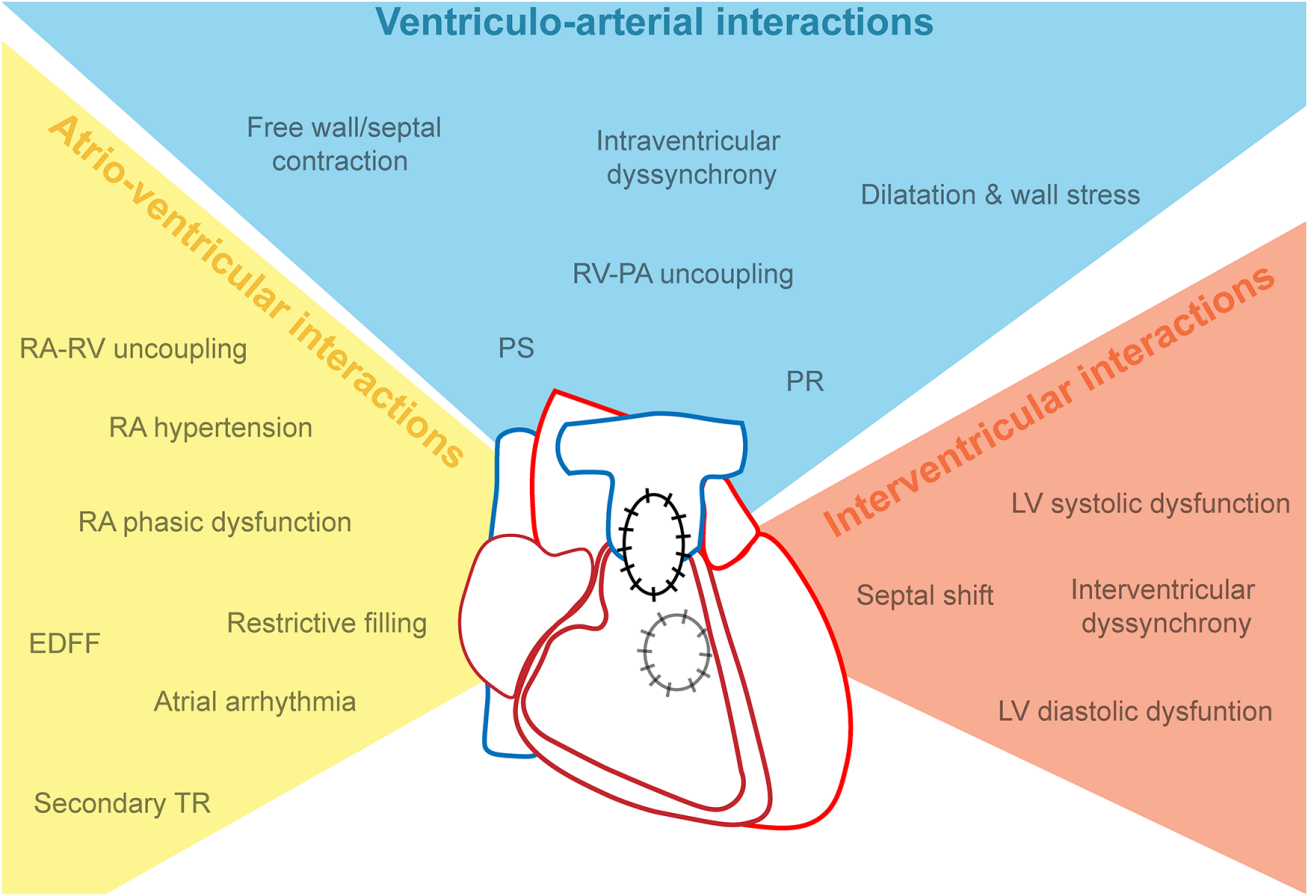
The syndrome of cardiac dysfunction in repaired tetralogy of Fallot, categorized as atrio-ventricular, ventriculo-arterial and interventricular interactions. EDFF, end-diastolic forward flow; LV, left ventricle; PA, pulmonary artery; PS, pulmonary stenosis; PR, pulmonary regurgitation; RA, right atrium; RV, right ventricle.

The temporal profile of RV adaptation to RV failure has multiple features, of which the causality is difficult to define. RV dilatation and prolonged QRS duration have been first recognized as hallmarks. Features of (mal)adaptation of the RV may be categorized into three groups: factors affecting ventriculo-arterial interactions (systolic function, PR), factors affecting atrio-ventricular interactions (e.g., impaired relaxation, atrial dysfunction), and factors affecting interventricular interactions (e.g., dyssynchrony, septal shift) (Figure 2).

### 3.1. Systolic dysfunction and ventriculo-arterial interactions

RV dilatation has been recognized as hallmark of RV dysfunction in pulmonary hypertension (16). In patients with rTOF with PR, progressive RV dilatation is a response to the PR-induced volume overload. Although (progressive) RV dilatation is widely used in guidelines for timing of pulmonary valve replacement, this strategy does not always ensure a favorable long-term outcome (11, 17, 18). In addition, longitudinal follow-up has demonstrated that RV dilatation is an unreliable marker of adverse events (19–22). Dilatation can contribute to development of secondary tricuspid regurgitation and the majority of TOF patients present with at least mild tricuspid regurgitation when referred for pulmonary valve replacement (23). Especially in the basal and mid-ventricular portions of the RV, geometrical changes of the tricuspid valve apparatus cause leaflet malcoaptation due to annular dilatation and papillary muscle displacement (24). Tricuspid regurgitation will lead to additional RV volume overload and might not necessarily regress after pulmonary valve replacement without concomitant tricuspid valve intervention (25).

RV dilatation in response to pressure overload, e.g., in pulmonary hypertension, is a hallmark of decompensation due to failure of homeometric adaptation (26). According to the law of Laplace, the larger inner diameter will increase wall stress (and thus afterload) if wall thickness and transmural pressure remain constant. In rTOF, RV dilatation can be accompanied by reduced ejection fraction signaling RV systolic dysfunction (27–29). However, decreases in RV ejection fraction over time are small and occur slowly, making it not an ideal marker for disease progression (19, 21). Maximal elastance, the load-independent assessment of contractility, remains the gold standard for systolic function, but data from invasive studies in rTOF are scarce and none of those studies compared rTOF patients to healthy controls (30, 31). Experimental models of chronic PR show a decrease in load-independent measures of contractility, such as maximal elastance and preload recruitable stroke work, which is an important distinction from the increased contractility observed in response to pressure overload (32, 33). Additionally, surgical RVOT scarring due to transannular patching significantly impairs RV contractility even further, due to RVOT aneurysm and akinesia (27, 34). Regional dysfunction might also be attributed to electromechanical dyssynchrony resulting in mechanical inefficiency of myocardial contraction (35).

**Figure 3:**
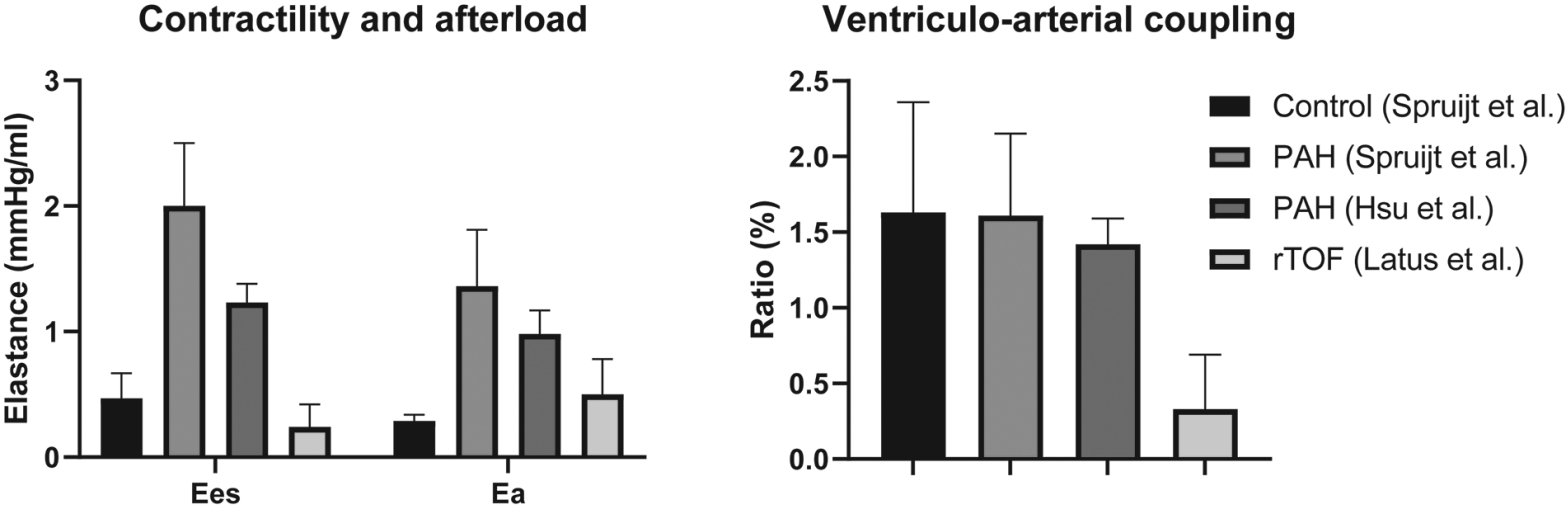
Comparison of contractility, afterload and ventriculo-arterial coupling in patients with pulmonary arterial hypertension and repaired tetralogy of Fallot. Ea, effective arterial elastance; Ees, end-systolic elastance; PAH, pulmonary arterial hypertension; rTOF, repaired tetralogy of Fallot. Spruijt et al. (39); Hsu et al. (38); Latus et al. (30).

Non-invasive markers of systolic function, e.g., RV ejection fraction, tricuspid annular plane systolic excursion and strain, are heavily load-dependent and a reflection of ventriculo-arterial coupling rather than contractility (36). Ventriculo-arterial coupling is the relation of contractility to pulmonary arterial afterload, an increasingly popular concept for assessment of RV systolic function. In pulmonary hypertension, deterioration of ventriculo-arterial coupling is a prelude to RV failure (37). As of date, its role in TOF remains unclear. Invasive assessment of ventriculo-arterial coupling and contractility has been performed only by Latus et al. (30). When comparing data of pulmonary hypertension patients with the study of Latus et al., shown in Figure 3, it appears that both contractility and afterload are much lower in rTOF, but the coupling ratio is decreased too (30, 38, 39). Of note, these different studies use different methods, so direct comparison is impossible. A recent study has published on ventriculo-arterial coupling in rTOF, non-invasively assessed by using cardiac magnetic resonance (CMR) derived RV volumes only (40). The need for pressures in the equation is supposedly eliminated by forcing the linear regression of the end-systolic pressure volume relation through the origin. Thereby, this method is nothing more than a variation on RV ejection fraction.

### 3.2. Diastolic dysfunction and atrio-ventricular interactions

**Figure 4:**
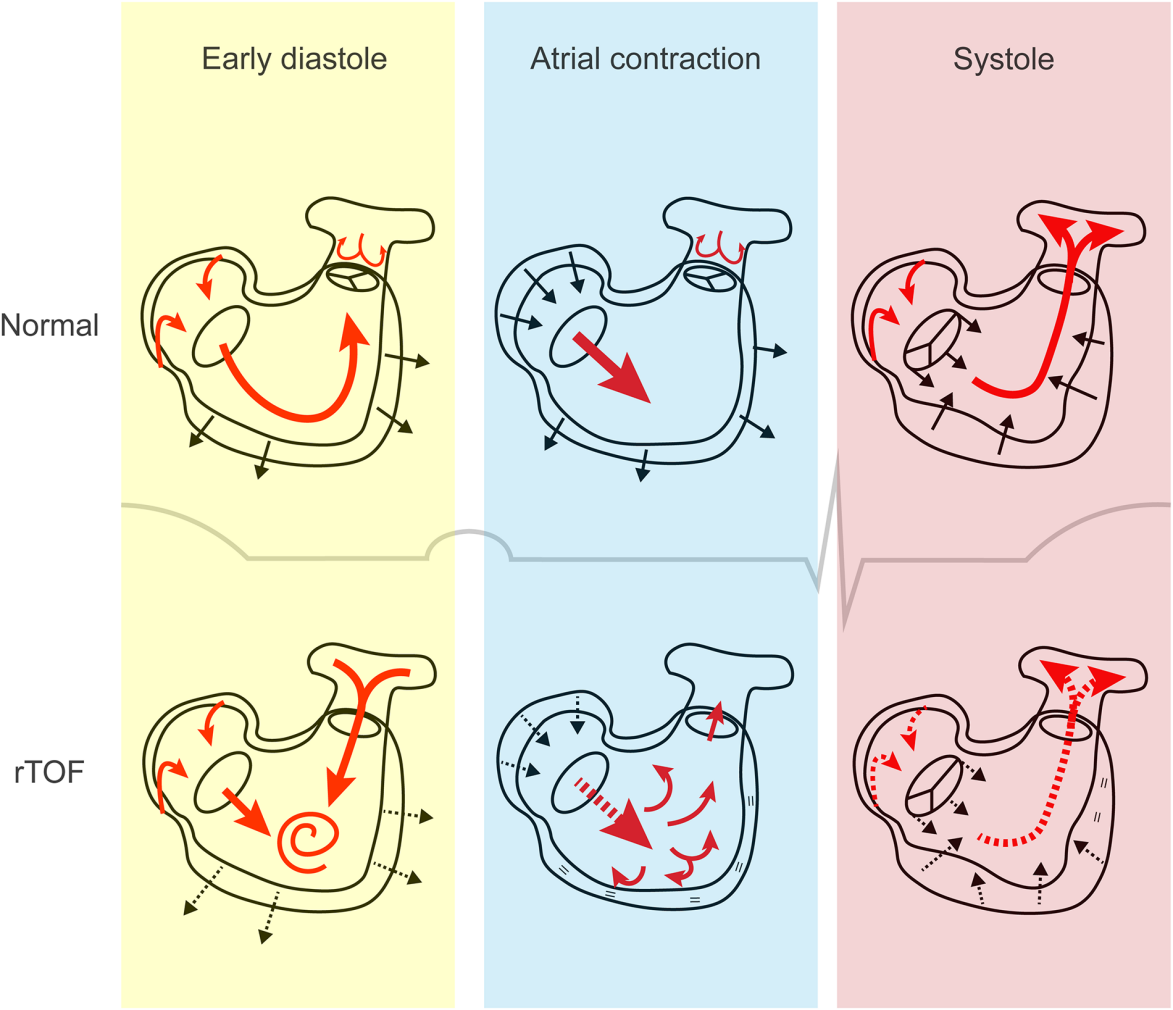
The cardiac cycle in tetralogy of Fallot. In early diastole, both right atrial conduit flow and pulmonary regurgitant flow contribute to right ventricular filling, resulting in increased flow vorticity. Decreased active relaxation and passive stiffness may be present. During atrial contraction, it is hypothesized that the stiff right ventricle may not be able to accommodate the additional blood volume, which gets displaced into the pulmonary artery. This phenomenon is referred to as end-diastolic forward flow. Right atrial contraction may be affected. In systole, decreased right ventricular contractility, dyssynchrony and outflow tract dysfunction can contribute to systolic dysfunction. Atrial reservoir filling is impaired if apical tricuspid movement is reduced. Red arrows, direction of blood flow; black arrows, movement of cardiac tissue (contraction or relaxation); dashed arrows, impaired flow or movement; equal sign, absent movement. rTOF, repaired tetralogy of Fallot.

Diastolic dysfunction might be an earlier, more prominent feature of maladaptation than systolic dysfunction. PR, the main hemodynamic lesion after corrective surgery, occurs during diastole (Figure 4). In the healthy RV, 4-dimensional flow measurements using CMR suggest that the inflow pattern allows an efficient outflow to the pulmonary artery during subsequent contraction. In rTOF patients with PR, however, abnormal diastolic flow patterns were consistently present, whereas systolic flow remained preserved in the large majority (41). From these studies it appears that PR flow interferes with normal diastolic vorticity enhancing RV filling from inflow to outflow, as is illustrated in Figure 4 (41, 42).

The purpose of diastole is to fill the ventricular cavity. Signs and symptoms associated with diastolic dysfunction include increased filling pressures, right atrial (RA) enlargement and peripheral edema. A general definition is lacking as several mechanisms may cause of diastolic dysfunction, such as impaired relaxation, increased stiffness or interventricular interactions. For this manuscript, diastolic dysfunction will be defined as the inability of the RV to fill in an energetically efficient manner. Normal diastolic function requires sufficient preload and low myocardial stiffness. Whereas preload in rTOF patients is generally maintained because of PR, it is not clear to what extent myocardial stiffness is affected. Invasive measurements in rTOF patients demonstrate increased RV end-diastolic pressures in half of the patients (43, 44). One study grouped pediatric and adult rTOF patients according to their indexed end-systolic volumes (cut-off 34.7 ml/m^2^) and found that patients with lower indexed end-systolic volumes also had higher myocardial stiffness, measured as load-independent end-diastolic elastance (31). In animal models of chronic PR, however, findings on end-diastolic elastance are conflicting, although a significantly higher stiffness was found in animals with pressure load-induced RV hypertrophy preceding the induction of PR (14, 45–47). In pressure overload, this increased stiffness as a result of reactive interstitial fibrosis is found only in more severe RV dysfunction (48). In isolated volume overload, however, interstitial fibrosis is not as commonly found (14, 46, 47, 49–51). Apart from fibrosis, myocardial stiffness is also determined by intrinsic stiffness of the cardiomyocytes, which mainly includes titin expression, its isoforms and phosphorylation, but also other sarcomeric and cytoskeletal proteins (52). Ex vivo studies on RV myocardium of rTOF patients demonstrated that myocardial and passive myofilament stiffness was not affected when compared to control samples (49, 53), which is in accordance with the observation that titin expression, isoforms and phosphorylation were unchanged in TOF patients (53–55). Other proteins have been associated with RV stiffness in TOF, mainly those involved in sarcomeric structure and calcium signaling, such as Z-disc proteins, tropomyosin-1 and phospholamban (54).

Unfortunately, demonstrating presence of diastolic dysfunction is complicated by the lack of reliable markers. Common measures of RV filling based on tricuspid inflow/annular velocity (*E*/*A*, *E*/*e*') do not correlate with RV end-diastolic pressure, and do not take the contribution of PR filling and increased diastolic vorticity into account (44, 56). Presence of end-diastolic forward flow during atrial contraction has been suggested to indicate reduced myocardial compliance during diastole, also referred to as “restrictive physiology”. Its role as a marker of dysfunction or poor prognosis is contested however (57–63).

Atrial function relates closely to diastolic ventricular function. Atrial functional measurements may therefore provide insights in the mechanisms of diastolic dysfunction and may serve as a disease marker, since robust alternatives are lacking. Increased RA pressures predict adverse cardiovascular events (64). RA phasic function has found to be altered in rTOF, with abnormalities in reservoir, conduit and pump function (Figure 4) (62, 65, 66). Load-independent atrio-ventricular coupling has not yet been investigated in TOF, but was found to be impaired in a model of early LV diastolic dysfunction and could serve as an early disease marker (67).

### 3.3. Interventricular interactions

In the assessment of RV systolic function, the role of the interventricular septum (IVS) is often underexplored relative to the RV free wall. The presence of the ventricular septal defect (VSD) divides the deep myocardial layer of the IVS, resulting in a reduction of longitudinally oriented myofibers (68, 69). Since 80% of the RV function is derived from longitudinal shortening, VSD-induced IVS dysfunction could add to global RV dysfunction. This type of dysfunction has received little attention (70). Interestingly, patients with isolated VSD show signs of impaired RV function late after VSD closure, but to what extent the IVS contributes to this dysfunction has yet to be determined (71, 72).

In recent years, it has become apparent that LV function is also affected in rTOF. LV diastolic dysfunction is commonly the result of geometrical changes and increased RV diastolic pressure, causing a leftward shift of the IVS hindering LV filling (46, 62, 73, 74). LV systolic dysfunction is found in 20% of rTOF patients and is independently associated with adverse outcomes (75, 76). Isolated PR does not seem to affect LV contractility *per se*, but electromechanical and interventricular dyssynchrony have been demonstrated as potential relevant mechanisms of biventricular dysfunction (77–79).

In summary, the phenotype of RV dysfunction can be regarded as a syndrome encompassing disturbed filling, dyssynchrony, and perhaps disturbed ventriculo-arterial coupling (Figure 2). The combination of abnormal diastolic flow patterns and reduced myocardial relaxation may lead to significant kinetic energy loss in diastole. In systole, lower contractility and dyssynchrony impairs efficient flow from the RV inlet into the pulmonary artery (41, 80, 81). A deeper understanding of the temporal relation and interactions between all these mechanisms is necessary to design specific treatments and preventive strategies.

## 4. Histopathology: what do we know?

Studies on histopathology of TOF are scarce. Consequently, much of what is known is derived from historical observations, mostly from patients corrected at what is presently regarded as “later stages”, e.g., more than 6 months of age or at death after uncorrected survival into adulthood (82–86). Hence, results may be affected by survival and selection bias, particularly in the group of patients studied during re-do surgery.

Most commonly described histopathological features are myocyte hypertrophy, fibrosis and altered capillary density. The degree of hypertrophy increases with age, likely initially as an adaptive response until a point of no return is reached with onset of maladaptive features (87–89). In a single-center study comparing biopsies obtained at primary repair between 4 months and 168 months of age, the degree of hypertrophy correlated with RV function, assessed as global strain. The recovery of RV strain after corrective surgery was reduced in the group of patients corrected after 1 year of age, suggesting irreversible remodeling if repair surgery is postponed (89). The hypertrophy is not homogenous; a postmortem study identified larger endocardial cardiomyocytes in the infundibulum as compared with other regions of the RV (90). In biopsies taken at a younger age less cardiomyocyte hypertrophy was found, which might be explained by the shorter exposure to overload (87–89). Alternatively, cardiomyocyte proliferation may be increased, enhancing RV adaptation. After birth, cardiomyocyte proliferation capacity is lost in mammals as a result of several postnatal processes, such as changes in oxygen levels and the transition from placental to enteral nutrition (91). However, recent studies have shown that cardiomyocytes retain the potential to proliferate after birth when under stress (12, 92).

Cardiomyocyte proliferation promotes adaptation to pressure overload better than cellular hypertrophy (92–95). Stimulating proliferation in mature cardiomyocytes or prolonging proliferative capacity after birth is therefore an attractive treatment target to preserve function of the overloaded RV. From cultured cardiomyocytes, isolated from biopsies of TOF patients, Liu et al. reported a high proportion of binucleated cardiomyocytes. This indicated that proliferation was initiated under adverse loading, but arrested in the cytokinesis stage. The authors demonstrated that failure of cytokinesis is mediated by beta-adrenergic signaling and that beta-adrenergic blockade can increase cell division by rescuing cardiomyocytes from cytokinesis failure. Additionally, this was confirmed in a neonatal mouse model of myocardial infarction (96). Although a previous randomized controlled trial (RCT) of beta-blockade in adult rTOF patients could not demonstrate a clinical benefit, a new RCT is being conducted to investigate its effects on cardiomyocyte proliferation in infants before repair surgery (ClinicalTrials.gov Identifier: NCT04713657) (9). Cell cycle activity can also be stimulated by administration of neuregulin-1. In both mature and weaning rats with RV pressure overload, neuregulin-1 attenuated maladaptive remodeling, with improved systolic and diastolic RV function (92, 94, 95). It is important to note that postnatal cardiomyocyte proliferation requires high oxidative energy metabolism (97). If proliferation was to be stimulated in an already metabolically demanding environment, this could exacerbate the energy deficit.

Myocardial fibrosis in rTOF has been associated with the risk of RV dysfunction, ventricular arrhythmia and sudden death (98–100). Recently, CMR studies in TOF have shown that increased late gadolinium enhancement, suggesting increased fibrosis, is a risk factor for late mortality (22). In this study, a composite fibrosis score was used, irrespective of location or type of fibrosis. Interstitial fibrosis, reactive to adverse loading, should be distinguished from replacement fibrosis following scarring, patch material and myocardial ischemia. Interstitial fibrosis has been shown in RV biopsies taken at the time of correction and appears to increase with the age of patients (83, 85, 87–89). Yet, according to RV samples of TOF patients younger than 9 months of age, collagen content is not significantly different from controls (101). These results suggest that neonatal RV pressure overload does not necessarily cause appreciable fibrosis in the first months of life, i.e., collagen production is still part of a beneficial adaptation process. Excessive collagen production being a feature mainly in patients older than 12 months at biopsy in the study of Xie et al. further supports this (89). Additionally, a greater amount of fibrosis was seen in patients with more functioning hypoxia inducible factor 1α (HIF1α) alleles, a mediator of the response to hypoxemia (102). Intriguingly, not all tissue biopsy studies of rTOF patients identified increased fibrosis (49, 87, 103). Assessing fibrosis in rTOF patients late after repair should be done in the context of sequential loading. Irrespective of the duration and degree of PR or residual RVOT obstruction, every rTOF patient was subjected to a period of neonatal pressure overload and underwent corrective surgery with aortic-cross clamping. Prolonged exposure to pressure overload can result in increased reactive fibrosis (48). Prolonged aortic-cross clamping time carries the risk of myocardial injury which will also result in fibrosis, but of the replacement type (104). Unlike in pressure overload, reactive interstitial fibrosis in response to isolated volume overload is much less pronounced or even absent (14, 34, 46, 47, 51, 105). In an older rTOF population with larger RV volumes, Yamamura et al. found that median collagen content of patients undergoing pulmonary valve replacement was 7.1%, which is comparable to the reference value of 7.4% found in healthy RV myocardium (103, 106). A subpopulation in the uppermost quartile of fibrosis in Yamamura's study had a median collagen content of 17.9%. In this small sample, the increased amount of fibrosis was associated with longer cross-clamp time as well as post-operative retention of larger RV end-systolic volumes, RV mass and RA area (103). In a younger, more contemporary managed rTOF population, fibrosis was not significantly different from control tissue samples (49). From these observations, it may be hypothesized that interstitial fibrosis presented in rTOF patients with PR is predominantly the remnant of pre-repair pressure overload and perhaps ischemic remodeling due to hypoxemia and aortic-cross clamping. The association between fibrosis and outcome may suggest a causal relationship, yet, inhibition of reactive fibrosis to either pressure or volume overload did not improve RV dysfunction (107, 108). In addition, interfering with fibrosis in cardiac tissue may have detrimental effects as fibrosis also has a cardioprotective effect (109). In line with this protective function, patients with more functional HIF1α alleles and more fibrosis benefited with less progression of PR, preservation of RV volumes and systolic function and greater freedom from re-interventions (102). These findings suggest that aforementioned adverse outcomes in rTOF patients could be related more to maladaptive fibrosis induced by triggers other than adverse loading. Reactive fibrosis, resulting from pressure and/or volume overload, appears to be merely a marker and not a targetable substrate. Further studies are necessary to identify the nature and optimal balance of adaptive and maladaptive fibrotic remodeling. At present, prevention of fibrosis by early repair and limiting formation of replacement fibrosis, e.g., by optimizing surgical technique and myocardial protection, seem superior to the use of anti-fibrotic treatments.

In addition to hypertrophy and fibrosis, increased capillary density has been demonstrated, both in tissue obtained during initial repair and in human heart specimens (86–88). The increased capillary density measured was attributed to an increased smaller number of vessels, with upregulation of pro-angiogenic factors. These findings indicate angiogenic sprouting to preserve the aerobic metabolic demand, although these vessels have been suggested to be immature and may not be conducting blood (87, 88). In contrast, in many (but not all) experimental models, RV pressure overload is associated with capillary rarefaction and a decrease in angiogenesis has been found to mark the transition from compensated to decompensated RV hypertrophy (110, 111). Based upon these observations, the increased angiogenic response in TOF is different from what is seen in acquired pulmonary hypertension, where cardiomyocyte hypertrophy exceeds the capacity for new capillary formation. Although the vasculature in TOF may not be completely functional, increased angiogenesis seems to be an adaptive feature. A role of hypoxemia and HIF1α in this process is likely, which is supported by the cardioprotective effects of the amount of functioning HIF1α alleles (102, 112). Several studies found increased mRNA expression of angiogenic factors, e.g., vascular endothelial growth factor (VEGF), in TOF patients at the time of repair when compared to healthy and VSD controls (87, 88, 102). However, cyanosis in TOF patients was associated with reduced VEGF expression when compared to those without cyanosis, complicating the interpretation of these angiogenic signals (55). Furthermore, a transcriptomic study demonstrated higher levels of apoptosis associated genes and decreased expression of genes associated with contractility and calcium handling in cyanotic patients (113). In order to preserve adequate oxygen delivery, myocardial vessel formation could be stimulated by targeting angiogenesis. Therapeutic options for promoting angiogenesis in the RV have recently been reviewed elsewhere (114).

In short, the main histopathological findings are cardiomyocyte hypertrophy and increased fibrosis, the severity increasing with older age at repair. Although cardiomyocyte proliferation is temporarily sustained in neonatal models of pressure overload, there is no concrete evidence of increased proliferation in TOF patients. This might suggest that by the time of repair, the adaptive response favors cardiomyocyte hypertrophy rather than proliferation. Late after repair, increased reactive fibrosis due to adverse loading is not necessarily a sign of maladaptation and may not be a suitable target for treatment. A remarkable distinction from pulmonary hypertension and isolated pressure overload is the increased, but potentially incomplete angiogenesis, which requires further investigation.

## 5. Is there a molecular signature in the right ventricle of tetralogy of Fallot?

**Figure 5:**
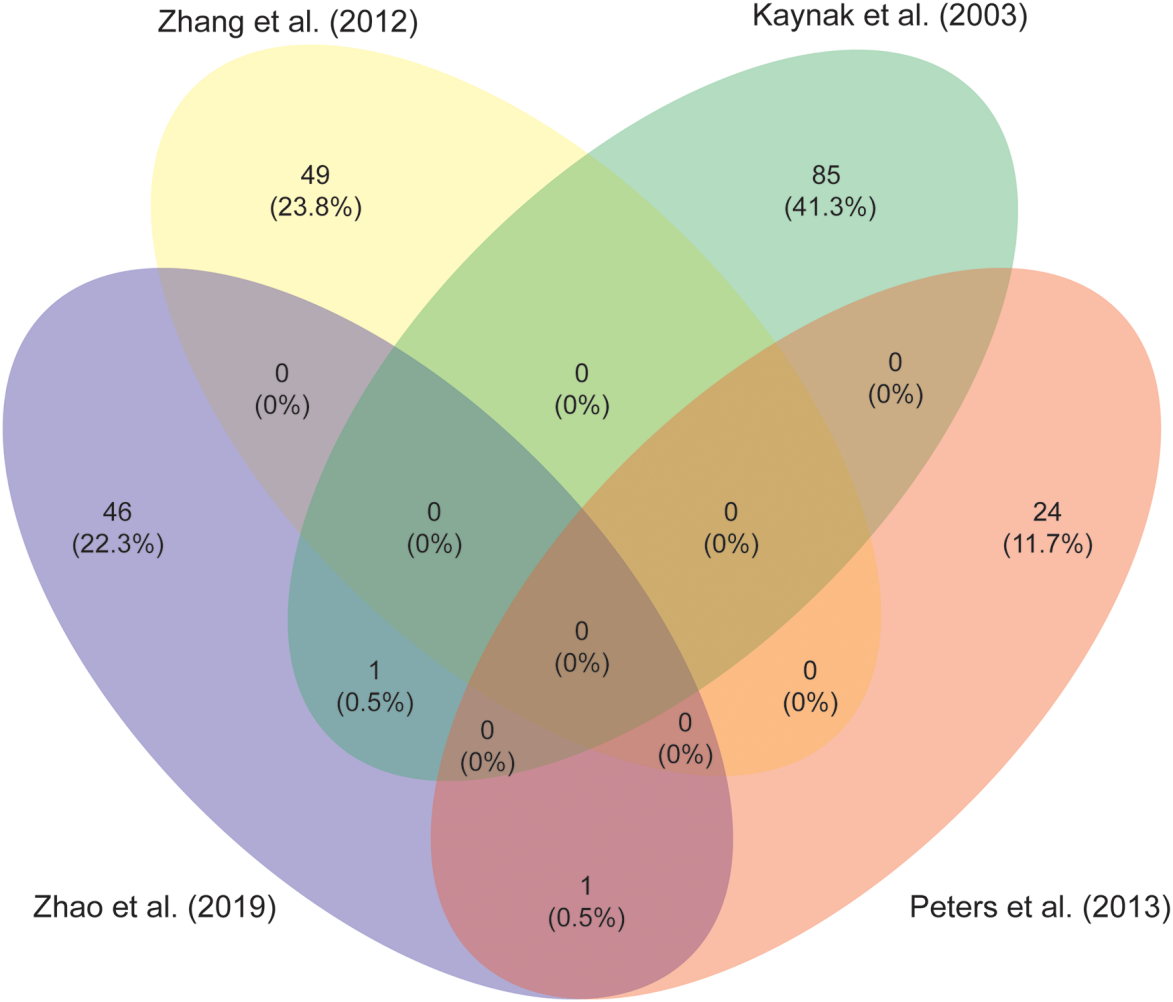
A Venn diagram to illustrate the overlap in differentially expressed genes in right ventricular myocardium from studies comparing tetralogy of Fallot patients to controls (88, 115-117).

Identification of pathways involved in RV remodeling is perhaps one of the most important steps in the search for specific treatments. Stimulation of adaptive features and inhibiting maladaptive remodeling might be key in preventing or delaying progression to RV failure. Unfortunately, a comprehensive analysis of patients tissue characteristics is complicated, as tissues may generally only be harvested at initial repair or at pulmonary valve replacement and are compared with different control groups. Intriguingly, there is little overlap in responses described in the studies aiming to elucidate adaptative pathways, as is illustrated in Figure 5, which shows a comparison of the studies investigating differential expression of mRNA between RV tissue samples of TOF patients and healthy controls (88, 115–117). Functional protein association network analysis of all the genes reported in these studies reveals several clusters, the largest encompassing extracellular matrix (ECM) proteins, fibroblasts and angiogenesis (118). Other clusters comprise protein synthesis, mitochondrial metabolism, second messengers, contractile apparatus and cell cycle associated proteins (see Supplementary Figure S1 and Table S1). There is ongoing debate on the contribution of inflammation in the development of heart failure (119). The majority of studies report changes in ECM proteins, which is linked to inflammatory pathways, yet, specific evidence of increased inflammation in the RV of TOF patients is lacking (49, 55, 87, 88, 113, 115–117, 120, 121).

Expression levels of ECM proteins vary between studies in tissue samples taken at the time of repair (54, 121). At the time of pulmonary valve replacement, however, no changes in ECM protein levels were found when comparing to healthy age-matched controls, although mRNA expression of several proteins involved in ECM are increased (49). Similarly, protein and mRNA expression of the ECM proteoglycan lumican was decreased at initial repair, but at the time of pulmonary valve replacement protein levels were unaltered despite increased mRNA expression (49, 121). Interestingly, in an animal model of PR, expression of ECM components also undergo a change in expression, from being initially downregulated to becoming upregulated in the chronic phase (105).

Proteins associated with calcium handling and the contractile machinery of the sarcomere have been found to be affected in uncorrected TOF patients when compared to isolated VSD patients (54). This is also evident from experimental PR models, with myosin heavy chain isoform switch as part of the fetal gene reactivation and altered expression of cardiac muscle actin and Xin actin binding protein for example (105, 122). Whether protein changes in the contractile apparatus are relevant in chronic volume overload late after TOF repair is questioned by the study of Brayson et al. (49). They found that in RV tissue taken at the time of pulmonary valve replacement, myofilament contractility and calcium sensitivity was comparable to non-diseased RV tissue. Enhancement of contractility could be managed by administration of myosin modulators. Omecamtiv mecarbil increases the amount of time in contraction and is investigated extensively in LV failure (123). Studies on its effects in the RV are still scarce however (124, 125). Interestingly, a rat model of chronic aortic regurgitation demonstrated that treatment with omecamtiv mecarbil significantly reduced wall stress in the LV (126). If this also applies to the RV, omecamtiv mecarbil could counteract adverse remodeling in TOF patients with PR. Although controversial, it is important to keep in mind potential side effects of these inotropic treatments. A metabolic burden could be imposed on the myocardium by increasing its oxygen consumption, while reducing myocardial perfusion by decreasing the time spent in diastole (127–130).

Cardiac metabolic inflexibility is the reliance on glycolysis alone for energy production instead of utilizing several different substrates. It has been suggested as a maladaptive phenomenon in a protein analysis study of RV tissue of TOF patients compared to other CHD diagnoses (121). This metabolic shift, with downregulation of beta-oxidation in favor of less efficient glycolysis, is already known from experimental RV pressure overload studies, but has also been observed in a model of chronic PR (105, 131, 132). Accommodating a higher metabolic demand requires optimizing myocardial energetics in the dysfunctional RV to prevent progression to failure. The metabolic shift towards inefficient glycolysis can be (partially) reversed. As an example, dichloroacetate enhances glucose oxidation, thereby restoring mitochondrial function and reducing myocardial apoptosis. Treatment in experimental models of pulmonary hypertension resulted in improved RV systolic function and reverse remodeling (133–135). Similar beneficial effects were observed during treatments to either increase or partially inhibit fatty acid beta oxidation, the latter in order to favor glucose oxidation (136, 137).

A recent study aimed to generate a circRNA-miRNA-mRNA network in TOF. They analyzed three datasets available in GEO databases comparing profiles and identified 29 miRNA, 13 circRNAs and 88 mRNAs (138). Interestingly, a comparison between overlapping miRNA profiles in four other studies yielded two miRNAs that were identified in all 4 studies and 5 miRNAs that were identified in at least 3 of the 4 studies (Table 1) (139–142). MiR-222, also identified in the network by Kan et al. has been associated with exercise-induced myocardial adaptation, atrial fibrillation, is involved in function of L-type calcium channels and is a target of angiogenesis and proliferation *via* ERBB4 and HIF1α (138, 143–146). MiR-194 has been linked to the p53 pathway and may be involved in pulmonary angiogenesis (147). For disease markers as well as therapeutic targets, these pathways may be worthwhile to explore, albeit that these have not been described in experimental models mimicking TOF.

**Table 1:** Heat map of overlapping differentially expressed microRNA in tetralogy of Fallot patients, as fold change compared to controls.

Hsa-miR nr.	Abu Halima et al. (139)	Liang et al. (140)	O’Brien et al. (141)	Zhang et al. (142)
222	−2.16	2.05	3.49	2.14
194	−2.49	1.24	2.16	1.84
93		1.23	1.65	2.55
155		2.02	1.68	2.37
381		−1.28	−0.16	2.32
30	−2.15	−1.32	1.65	
151	−2.20	1.11	1.14	

Generally, experimental studies show that many molecular mechanisms of RV adaptation are similar in pressure and volume overload, albeit that the latter exhibits a milder phenotype with slower disease progression (50, 148). In addition, most clinical studies are too heterogeneous to compare in order to derive a disease-specific molecular signature. Also, the relation between tissue expression patterns and serum biomarkers is difficult to determine. A comprehensive analysis of serum biomarkers associated with adverse outcomes could provide additional insights in adaptation of the overloaded RV, but is beyond the scope of this review (149).

## 6. Current and future strategies

### 6.1. Treatment of right ventricular dysfunction

Currently, attempts for medical support of the failing RV in rTOF have been futile (3, 150). A widely accepted treatment regimen, including cornerstone medication, resynchronization and exercise for cardiac rehabilitation, as described in patients with heart failure due to LV dysfunction, is therefore absent. Guidelines on medical therapy are limited to diuretics, merely for relieving symptoms of RV decompensation (151). Furthermore, the lack of robust clinical markers to assess RV dysfunction and outcomes in rTOF patients complicates the decision regarding the initiation of treatment.

As a cornerstone of heart failure therapy, renin-angiotensin-aldosterone system (RAAS) inhibition lowers systemic afterload and blood volume status, whilst having antifibrotic and antihypertrophic effects in the myocardium itself (152). Several RCTs on RAAS inhibition have been conducted in TOF, but none could demonstrate a beneficial effect on RV function (8, 153). Although there is evidence of RAAS activation in the setting of RV pressure overload, its mechanisms in TOF have yet to be elucidated (108, 154, 155). The novel valsartan-sacubitril combination drug inhibits the angiotensin receptor-neprilysin pathway and might be promising in the treatment of RV dysfunction (156). Currently only a case report of the use of valsartan-sacubitril in TOF is available, but in a small cohort of patients with a systemic RV improvement of echocardiographic markers and reduction of NT-pro-BNP was observed over six month follow-up (157, 158).

Sodium-glucose cotransporter-2 (SGLT2) inhibitors are increasingly used in the treatment of LV failure. Even though the mechanism of action is still unclear and there are no myocardial SGLT2 receptors, they lower cardiovascular mortality independent of patient diabetic status (159). From experimental studies in rats with monocrotalin induced pulmonary hypertension, it appears that SGLT2 inhibitors lower pulmonary pressure by attenuating pulmonary vascular remodeling (160, 161). When regarding effects on the RV itself, however, dapagliflozin treatment did not result in any improvements in myocardial function or remodeling (162). Clinical data on primary RV dysfunction is lacking except for a case report in systemic RV (163).

Rather than ameliorating adverse loading, enhancing myocardial contractility is another potential treatment path. Phosphodiesterase 5 (PDE5) inhibitors are known for their pulmonary vasodilatory effects in the treatment of pulmonary hypertension. Recently, they have been found to also increase RV contractility (133, 164). In RV pressure overload models, PDE5 becomes upregulated in remodeled RV myocardium and PDE5 inhibition has resulted in a multitude of effects, independent of afterload (165). Besides its RV specific inotropic effects, improvement of diastolic dysfunction and dilatation have been observed in sildenafil-treated rats with pulmonary artery banding (166). The improvement in diastolic function might be explained by the increased phosphorylation of titin to its more distensible form (167). As discussed earlier, however, the involvement of titin in diastolic dysfunction in rTOF has yet to be demonstrated.

Important gain of function can be achieved by improving the efficiency of RV contraction in TOF. Electromechanical dyssynchrony and right bundle branch physiology hinder a properly coordinated contraction, which makes rTOF a “synchronopathy” (35). The studies of Janousek et al. on cardiac resynchronization therapy of the RV in CHD demonstrated improved mechanics of contraction in both acute and chronic phases (78, 168). Patch material and scarring in the RVOT and VSD, however, remain akinetic regions where loss of energy and efficiency are bound to occur. Removal of dysfunctional RVOT tissue at the time of pulmonary valve replacement did not seem to have benefits over isolated pulmonary valve replacement (169). Tissue engineering might be able to overcome the problems arising from the electromechanical disconnection of patch material and the surrounding scar formation. Patches could be biological scaffolds incorporating cardiomyocytes or endothelial cells to become functional myocardium or vessel respectively (170). With tissue engineering, other opportunities may arise, like implantation of contractile bands to enhance RV biomechanics (171).

### 6.2. Preventing right ventricular deterioration

Patients with CHD are usually in follow-up from birth. This provides a major opportunity for implementing preventive strategies early on in the disease process, in contrast to acquired heart failure which is only diagnosed and treated in a symptomatic stage.

The obvious solution to prevent RV dysfunction would be the relief of adverse loading as soon as possible. Neonatal correction minimizes the duration of pressure overload and hypoxemia, but at the cost of a higher risk of more severe residual lesions (i.e., PR and RVOT obstruction) and worse event-free survival (172). Furthermore, the impact on RV function and histological changes is limited if correction is performed before the first year of age (87, 89). This justifies an early, but not neonatal, repair strategy to address the repercussions of loading on remodeling. Chronic PR will probably remain a culprit for as long as it takes for growing tissue valves to become available. Until then, treatment strategies could/should focus on reinforcing adaptive processes of RV remodeling in order to better accommodate adverse loading (92, 96).

In addition, exercise training could aid in strengthening the RV. Exercise training has already proven to be useful in the treatment of left-sided heart failure (173, 174). Intriguingly, recent experimental studies have shown that exercise also induces cardiomyocyte proliferation (175, 176). In the treatment of RV dysfunction in CHD, studies have demonstrated the safety of exercise therapy and applicability even in young children (177–182). Although significant improvements in cardiac function have not yet been confirmed in rTOF cohorts, several investigations are ongoing and looking into optimal training regimens (177, 178, 181, 182).

## 7. Summary

TOF is characterized by a unique sequential loading pattern. Histopathology reflects these different stages of loading, but at present does not provide a targetable substrate for therapy. There is little to no consensus regarding specific adaptation mechanisms due to the wide variation in studies, but it seems likely there is no unifying molecular pathway leading to dysfunction. Knowledge of the distinctive physiology in TOF explains the lack of success of current treatment strategies. Cardiac dysfunction can be described as a combination of impaired ventricular kinetic energy and a disturbed filling pattern. The potential for future strategies is in optimizing RV biomechanics, kinetic energy and treatments to improve myocardial relaxation. In addition to novel drug targets, other promising options include resynchronization therapy, tissue engineered grafts and strategies aimed to strengthen the RV.

## References

[1] van der Bom T Bouma BJ Meijboom FJ Zwinderman AH Mulder BJ. The prevalence of adult congenital heart disease, results from a systematic review and evidence based calculation. Am Heart J. (2012) 164(4):568–75. 10.1016/j.ahj.2012.07.023 23067916

[2] Engelfriet P Boersma E Oechslin E Tijssen J Gatzoulis MA Thilen U The spectrum of adult congenital heart disease in Europe: morbidity and mortality in a 5 year follow-up period. The euro heart survey on adult congenital heart disease. Eur Heart J. (2005) 26(21):2325–33. 10.1093/eurheartj/ehi396 15996978

[3] Stout KK Broberg CS Book WM Cecchin F Chen JM Dimopoulos K Chronic heart failure in congenital heart disease: a scientific statement from the American heart association. Circulation. (2016) 133(8):770–801. 10.1161/CIR.0000000000000352 26787728

[4] van der Ven JPG van den Bosch E Bogers A Helbing WA. Current outcomes and treatment of tetralogy of Fallot. F1000Res. (2019) 8:F1000. 10.12688/f1000research.17174.1. 31508203 PMC6719677

[5] Karamlou T McCrindle BW Williams WG. Surgery insight: late complications following repair of tetralogy of Fallot and related surgical strategies for management. Nat Clin Pract Cardiovasc Med. (2006) 3(11):611–22. 10.1038/ncpcardio0682 17063166

[6] Gurvitz M Burns KM Brindis R Broberg CS Daniels CJ Fuller SM Emerging research directions in adult congenital heart disease: a report from an NHLBI/ACHA working group. J Am Coll Cardiol. (2016) 67(16):1956–64. 10.1016/j.jacc.2016.01.062 27102511 PMC4846980

[7] Bouzas B Kilner PJ Gatzoulis MA. Pulmonary regurgitation: not a benign lesion. Eur Heart J. (2005) 26(5):433–9. 10.1093/eurheartj/ehi091 15640261

[8] Bokma JP Winter MM van Dijk AP Vliegen HW van Melle JP Meijboom FJ Effect of losartan on right ventricular dysfunction: results from the double-blind, randomized REDEFINE trial (right ventricular dysfunction in tetralogy of Fallot: inhibition of the renin-angiotensin-aldosterone system) in adults with repaired tetralogy of Fallot. Circulation. (2018) 137(14):1463–71. 10.1161/CIRCULATIONAHA.117.031438 29222139

[9] Norozi K Bahlmann J Raab B Alpers V Arnhold JO Kuehne T A prospective, randomized, double-blind, placebo controlled trial of beta-blockade in patients who have undergone surgical correction of tetralogy of Fallot. Cardiol Young. (2007) 17(4):372–9. 10.1017/S1047951107000844 17572925

[10] Bokma JP Geva T Sleeper LA Babu Narayan SV Wald R Hickey K A propensity score-adjusted analysis of clinical outcomes after pulmonary valve replacement in tetralogy of Fallot. Heart. (2018) 104(9):738–44. 10.1136/heartjnl-2017-312048 29092913

[11] Mongeon FP Ben Ali W Khairy P Bouhout I Therrien J Wald RM Pulmonary valve replacement for pulmonary regurgitation in adults with tetralogy of Fallot: a meta-analysis-A report for the writing committee of the 2019 update of the Canadian cardiovascular society guidelines for the management of adults with congenital heart disease. Can J Cardiol. (2019) 35(12):1772–83. 10.1016/j.cjca.2019.08.031 31813508

[12] Ding X Wang S Wang Y Yang J Bao N Liu J Neonatal heart responds to pressure overload with differential alterations in Various cardiomyocyte maturation programs that accommodate simultaneous hypertrophy and hyperplasia. Front Cell Dev Biol. (2020) 8:596960. 10.3389/fcell.2020.596960 33330485 PMC7710899

[13] Bertranou E Blackstone E Hazelrig J Turner M Kirklin J. Life expectancy without surgery in tetralogy of Fallot. Am J Cardiol. (1978) 42(3):458–66. 10.1016/0002-9149(78)90941-4 685856

[14] Bove T Vandekerckhove K Bouchez S Wouters P Somers P Van Nooten G. Role of myocardial hypertrophy on acute and chronic right ventricular performance in relation to chronic volume overload in a porcine model: relevance for the surgical management of tetralogy of Fallot. J Thorac Cardiovasc Surg. (2014) 147(6):1956–65. 10.1016/j.jtcvs.2013.10.026 24280710

[15] Oosterhof T Meijboom FJ Vliegen HW Hazekamp MG Zwinderman AH Bouma BJ Long-term follow-up of homograft function after pulmonary valve replacement in patients with tetralogy of Fallot. Eur Heart J. (2006) 27(12):1478–84. 10.1093/eurheartj/ehl033 16707545

[16] van Wolferen SA Marcus JT Boonstra A Marques KM Bronzwaer JG Spreeuwenberg MD Prognostic value of right ventricular mass, volume, and function in idiopathic pulmonary arterial hypertension. Eur Heart J. (2007) 28(10):1250–7. 10.1093/eurheartj/ehl477 17242010

[17] Geva T Mulder B Gauvreau K Babu-Narayan SV Wald RM Hickey K Preoperative predictors of death and sustained ventricular tachycardia after pulmonary valve replacement in patients with repaired tetralogy of Fallot enrolled in the INDICATOR cohort. Circulation. (2018) 138(19):2106–15. 10.1161/CIRCULATIONAHA.118.034740 30030416

[18] Van den Eynde J Sa M Vervoort D Roever L Meyns B Budts W Pulmonary valve replacement in tetralogy of Fallot: an updated meta-analysis. Ann Thorac Surg. (2022) 113(3):1036–46. 10.1016/j.athoracsur.2020.11.040 33378694

[19] Luijnenburg SE Helbing WA Moelker A Kroft LJ Groenink M Roos-Hesselink JW 5-year Serial follow-up of clinical condition and ventricular function in patients after repair of tetralogy of Fallot. Int J Cardiol. (2013) 169(6):439–44. 10.1016/j.ijcard.2013.10.013 24182670

[20] Rutz T Ghandour F Meierhofer C Naumann S Martinoff S Lange R Evolution of right ventricular size over time after tetralogy of Fallot repair: a longitudinal cardiac magnetic resonance study. Eur Heart J Cardiovasc Imaging. (2017) 18(3):364–70. 10.1093/ehjci/jew273 28363200

[21] Wald RM Valente AM Gauvreau K Babu-Narayan SV Assenza GE Schreier J Cardiac magnetic resonance markers of progressive RV dilation and dysfunction after tetralogy of Fallot repair. Heart. (2015) 101(21):1724–30. 10.1136/heartjnl-2015-308014 26276804

[22] Ghonim S Gatzoulis MA Ernst S Li W Moon JC Smith GC Predicting survival in repaired tetralogy of Fallot: a lesion-specific and personalized approach. JACC Cardiovasc Imaging. (2022) 15(2):257–68. 10.1016/j.jcmg.2021.07.026 34656466 PMC8821017

[23] Mahle WT Parks WJ Fyfe DA Sallee D. Tricuspid regurgitation in patients with repaired tetralogy of Fallot and its relation to right ventricular dilatation. Am J Cardiol. (2003) 92(5):643–5. 10.1016/S0002-9149(03)00746-X 12943899

[24] Topilsky Y Khanna A Le Tourneau T Park S Michelena H Suri R Clinical context and mechanism of functional tricuspid regurgitation in patients with and without pulmonary hypertension. Circ Cardiovasc Imaging. (2012) 5(3):314–23. 10.1161/CIRCIMAGING.111.967919 22447806

[25] Van den Eynde J Callahan CP Lo Rito M Hussein N Carvajal H Guariento A Tricuspid valve intervention at the time of pulmonary valve replacement in adults with congenital heart disease: a systematic review and meta-analysis. J Am Heart Assoc. (2021) 10(24):e022909. 10.1161/JAHA.121.022909 34873914 PMC9075262

[26] Naeije R Brimioulle S Dewachter L. Biomechanics of the right ventricle in health and disease (2013 grover conference series). Pulm Circ. (2014) 4(3):395–406. 10.1086/677354 25621153 PMC4278599

[27] Davlouros PA Kilner PJ Hornung TS Li W Francis JM Moon JC Right ventricular function in adults with repaired tetralogy of Fallot assessed with cardiovascular magnetic resonance imaging: detrimental role of right ventricular outflow aneurysms or akinesia and adverse right-to-left ventricular interaction. J Am Coll Cardiol. (2002) 40(11):2044–52. 10.1016/S0735-1097(02)02566-4 12475468

[28] Bidviene J Muraru D Kovacs A Lakatos B Ereminiene E Liptai C Global and regional right ventricular mechanics in repaired tetralogy of Fallot with chronic severe pulmonary regurgitation: a three-dimensional echocardiography study. Cardiovasc Ultrasound. (2021) 19(1):28. 10.1186/s12947-021-00260-3 34362392 PMC8349004

[29] Morcos P Vick GW 3rd Sahn DJ Jerosch-Herold M Shurman A Sheehan FH. Correlation of right ventricular ejection fraction and tricuspid annular plane systolic excursion in tetralogy of Fallot by magnetic resonance imaging. Int J Cardiovasc Imaging. (2009) 25(3):263–70. 10.1007/s10554-008-9387-0 19048388

[30] Latus H Binder W Kerst G Hofbeck M Sieverding L Apitz C. Right ventricular-pulmonary arterial coupling in patients after repair of tetralogy of Fallot. J Thorac Cardiovasc Surg. (2013) 146(6):1366–72. 10.1016/j.jtcvs.2013.02.039 23507126

[31] Uebing A Fischer G Schlangen J Apitz C Steendijk P Kramer HH. Can we use the end systolic volume index to monitor intrinsic right ventricular function after repair of tetralogy of Fallot? Int J Cardiol. (2011) 147(1):52–7. 10.1016/j.ijcard.2009.07.031 19716612

[32] Borgdorff MA Dickinson MG Berger RM Bartelds B. Right ventricular failure due to chronic pressure load: what have we learned in animal models since the NIH working group statement? Heart Fail Rev. (2015) 20(4):475–91. 10.1007/s10741-015-9479-6 25771982 PMC4463984

[33] Bossers GPL Hagdorn QAJ Ploegstra MJ Borgdorff MAJ Sillje HHW Berger RMF Volume load-induced right ventricular dysfunction in animal models: insights in a translational gap in congenital heart disease. Eur J Heart Fail. (2018) 20(4):808–12. 10.1002/ejhf.931 28925007

[34] Bove T Bouchez S De Hert S Wouters P De Somer F Devos D Acute and chronic effects of dysfunction of right ventricular outflow tract components on right ventricular performance in a porcine model: implications for primary repair of tetralogy of fallot. J Am Coll Cardiol. (2012) 60(1):64–71. 10.1016/j.jacc.2012.03.035 22742402

[35] Hui W Slorach C Dragulescu A Mertens L Bijnens B Friedberg MK. Mechanisms of right ventricular electromechanical dyssynchrony and mechanical inefficiency in children after repair of tetralogy of fallot. Circ Cardiovasc Imaging. (2014) 7(4):610–8. 10.1161/CIRCIMAGING.113.001483 24785673

[36] Guihaire J Haddad F Boulate D Decante B Denault AY Wu J Non-invasive indices of right ventricular function are markers of ventricular-arterial coupling rather than ventricular contractility: insights from a porcine model of chronic pressure overload. Eur Heart J Cardiovasc Imaging. (2013) 14(12):1140–9. 10.1093/ehjci/jet092 23677917

[37] Vonk-Noordegraaf A Haddad F Chin KM Forfia PR Kawut SM Lumens J Right heart adaptation to pulmonary arterial hypertension: physiology and pathobiology. J Am Coll Cardiol. (2013) 62(25 Suppl):D22–33. 10.1016/j.jacc.2013.10.027 24355638

[38] Hsu S Houston BA Tampakakis E Bacher AC Rhodes PS Mathai SC Right ventricular functional reserve in pulmonary arterial hypertension. Circulation. (2016) 133(24):2413–22. 10.1161/CIRCULATIONAHA.116.022082 27169739 PMC4907868

[39] Spruijt OA de Man FS Groepenhoff H Oosterveer F Westerhof N Vonk-Noordegraaf A The effects of exercise on right ventricular contractility and right ventricular-arterial coupling in pulmonary hypertension. Am J Respir Crit Care Med. (2015) 191(9):1050–7. 10.1164/rccm.201412-2271OC 25710636

[40] Sandeep B Huang X Li Y Wang X Mao L Kan Y Evaluation of right ventricle pulmonary artery coupling on right ventricular function in post operative tetralogy of Fallot patients underwent for pulmonary valve replacement. J Cardiothorac Surg. (2020) 15(1):241. 10.1186/s13019-020-01281-1 32912248 PMC7487999

[41] Francois CJ Srinivasan S Schiebler ML Reeder SB Niespodzany E Landgraf BR 4D Cardiovascular magnetic resonance velocity mapping of alterations of right heart flow patterns and main pulmonary artery hemodynamics in tetralogy of Fallot. J Cardiovasc Magn Reson. (2012) 14:16. 10.1186/1532-429X-14-16 22313680 PMC3305663

[42] Kilner PJ Yang GZ Wilkes AJ Mohiaddin RH Firmin DN Yacoub MH. Asymmetric redirection of flow through the heart. Nature. (2000) 404(6779):759–61. 10.1038/35008075 10783888

[43] Egbe AC Pellikka PA Miranda WR Bonnichsen C Reddy YNV Borlaug BA Echocardiographic predictors of severe right ventricular diastolic dysfunction in tetralogy of Fallot: relations to patient outcomes. Int J Cardiol. (2020) 306:49–55. 10.1016/j.ijcard.2020.02.067 32145939 PMC7297267

[44] DiLorenzo M Hwang WT Goldmuntz E Ky B Mercer-Rosa L. Diastolic dysfunction in tetralogy of Fallot: comparison of echocardiography with catheterization. Echocardiography. (2018) 35(10):1641–8. 10.1111/echo.14113 30105757 PMC6205242

[45] Agger P Hyldebrandt JA Nielsen EA Hjortdal V Smerup M. A novel porcine model for right ventricular dilatation by external suture plication of the pulmonary valve leaflets–practical and reproducible. Interact Cardiovasc Thorac Surg. (2010) 10(6):962–6. 10.1510/icvts.2009.227264 20197345

[46] Kuehne T Saeed M Gleason K Turner D Teitel D Higgins CB Effects of pulmonary insufficiency on biventricular function in the developing heart of growing swine. Circulation. (2003) 108(16):2007–13. 10.1161/01.CIR.0000092887.84425.09 14557371

[47] Bove T Alipour Symakani R Verbeke J Vral A El Haddad M De Wilde H Study of the time-relationship of the mechano-electrical interaction in an animal model of tetralogy of Fallot: implications for the risk assessment of ventricular arrhythmias. Interact Cardiovasc Thorac Surg. (2020) 31(1):129–37. 10.1093/icvts/ivaa047 32243531

[48] Rain S Andersen S Najafi A Gammelgaard Schultz J da Silva Goncalves Bos D Handoko ML Right ventricular myocardial stiffness in experimental pulmonary arterial hypertension: relative contribution of fibrosis and myofibril stiffness. Circ Heart Fail. (2016) 9(7):e002636. 10.1161/CIRCHEARTFAILURE.115.002636 27370069 PMC4956674

[49] Brayson D Holohan SJ Bardswell SC Arno M Lu H Jensen HK Right ventricle has normal myofilament function but shows perturbations in the expression of extracellular matrix genes in patients with tetralogy of Fallot undergoing pulmonary valve replacement. J Am Heart Assoc. (2020) 9(16):e015342. 10.1161/JAHA.119.015342 32805183 PMC7660801

[50] Borgdorff MA Bartelds B Dickinson MG Steendijk P de Vroomen M Berger RM. Distinct loading conditions reveal various patterns of right ventricular adaptation. Am J Physiol Heart Circ Physiol. (2013) 305(3):H354–64. 10.1152/ajpheart.00180.2013 23729212

[51] Chemaly ER Kang S Zhang S McCollum L Chen J Benard L Differential patterns of replacement and reactive fibrosis in pressure and volume overload are related to the propensity for ischaemia and involve resistin. J Physiol. (2013) 591(21):5337–55. 10.1113/jphysiol.2013.258731 24018949 PMC3936371

[52] Villalobos Lizardi JC Baranger J Nguyen MB. A guide for assessment of myocardial stiffness in health and disease. Nat Cardiovasc Res. (2022) 1:8–22. 10.1038/s44161-021-00007-3 39196108

[53] Chaturvedi RR Herron T Simmons R Shore D Kumar P Sethia B Passive stiffness of myocardium from congenital heart disease and implications for diastole. Circulation. (2010) 121(8):979–88. 10.1161/CIRCULATIONAHA.109.850677 20159832

[54] Bond AR Iacobazzi D Abdul-Ghani S Ghorbel M Heesom K Wilson M Changes in contractile protein expression are linked to ventricular stiffness in infants with pulmonary hypertension or right ventricular hypertrophy due to congenital heart disease. Open Heart. (2018) 5(1):e000716. 10.1136/openhrt-2017-000716 29344379 PMC5761287

[55] Reddy S Osorio JC Duque AM Kaufman BD Phillips AB Chen JM Failure of right ventricular adaptation in children with tetralogy of Fallot. Circulation. (2006) 114(1 Suppl):I37–42. 10.1161/CIRCULATIONAHA.105.001248 16820602

[56] Hayabuchi Y Sakata M Ohnishi T Inoue M Kagami S. Ratio of early diastolic tricuspid inflow to tricuspid lateral annulus velocity reflects pulmonary regurgitation severity but not right ventricular diastolic function in children with repaired tetralogy of Fallot. Pediatr Cardiol. (2013) 34(5):1112–7. 10.1007/s00246-012-0612-1 23247587

[57] Gatzoulis MA Clark AL Cullen S Newman CG Redington AN. Right ventricular diastolic function 15 to 35 years after repair of tetralogy of Fallot. Restrictive physiology predicts superior exercise performance. Circulation. (1995) 91(6):1775–81. 10.1161/01.CIR.91.6.1775 7882487

[58] Kutty S Valente AM White MT Hickey K Danford DA Powell AJ Usefulness of pulmonary arterial End-diastolic forward flow late after tetralogy of Fallot repair to predict a “restrictive” right ventricle. Am J Cardiol. (2018) 121(11):1380–6. 10.1016/j.amjcard.2018.02.025 29678339

[59] Van den Eynde J Derdeyn E Schuermans A Shivaram P Budts W Danford DA End-Diastolic forward flow and restrictive physiology in repaired tetralogy of Fallot: a systematic review and meta-analysis. J Am Heart Assoc. (2022) 11(7):e024036. 10.1161/JAHA.121.024036 35301867 PMC9075485

[60] Helbing WA Niezen RA Le Cessie S van der Geest RJ Ottenkamp J de Roos A. Right ventricular diastolic function in children with pulmonary regurgitation after repair of tetralogy of Fallot: volumetric evaluation by magnetic resonance velocity mapping. J Am Coll Cardiol. (1996) 28(7):1827–35. 10.1016/S0735-1097(96)00387-7 8962573

[61] van den Berg J Wielopolski PA Meijboom FJ Witsenburg M Bogers AJ Pattynama PM Diastolic function in repaired tetralogy of Fallot at rest and during stress: assessment with MR imaging. Radiology. (2007) 243(1):212–9. 10.1148/radiol.2431060213 17293573

[62] Luijnenburg SE Peters RE van der Geest RJ Moelker A Roos-Hesselink JW de Rijke YB Abnormal right atrial and right ventricular diastolic function relate to impaired clinical condition in patients operated for tetralogy of Fallot. Int J Cardiol. (2013) 167(3):833–9. 10.1016/j.ijcard.2012.02.011 22390967

[63] Mercer-Rosa L Fogel MA Paridon SM Rychik J Yang W Goldmuntz E. Revisiting the End-diastolic forward flow (restrictive physiology) in tetralogy of Fallot: an exercise, echocardiographic, and magnetic resonance study. JACC Cardiovasc Imaging. (2018) 11(10):1547–8. 10.1016/j.jcmg.2018.01.008 29550323

[64] Egbe AC Bonnichsen C Reddy YNV Anderson JH Borlaug BA. Pathophysiologic and prognostic implications of right atrial hypertension in adults with tetralogy of Fallot. J Am Heart Assoc. (2019) 8(22):e014148. 10.1161/JAHA.119.014148 31701796 PMC6915294

[65] Riesenkampff E Mengelkamp L Mueller M Kropf S Abdul-Khaliq H Sarikouch S Integrated analysis of atrioventricular interactions in tetralogy of Fallot. Am J Physiol Heart Circ Physiol. (2010) 299(2):H364–71. 10.1152/ajpheart.00264.2010 20495149 PMC2930385

[66] Kutty S Shang Q Joseph N Kowallick JT Schuster A Steinmetz M Abnormal right atrial performance in repaired tetralogy of Fallot: a CMR feature tracking analysis. Int J Cardiol. (2017) 248:136–42. 10.1016/j.ijcard.2017.06.121 28712562

[67] Zakeri R Moulay G Chai Q Ogut O Hussain S Takahama H Left atrial remodeling and atrioventricular coupling in a canine model of early heart failure with preserved ejection fraction. Circ Heart Fail. (2016) 9(10):e003238. 10.1161/CIRCHEARTFAILURE.115.003238 27758811 PMC5082983

[68] Tous C Gentles TL Young AA Pontre BP. Ex vivo cardiovascular magnetic resonance diffusion weighted imaging in congenital heart disease, an insight into the microstructures of tetralogy of Fallot, biventricular and univentricular systemic right ventricle. J Cardiovasc Magn Reson. (2020) 22(1):69. 10.1186/s12968-020-00662-8 32951605 PMC7504600

[69] Sanchez-Quintana D Anderson RH Ho SY. Ventricular myoarchitecture in tetralogy of Fallot. Heart. (1996) 76(3):280–6. 10.1136/hrt.76.3.280 8868990 PMC484521

[70] Buckberg G Hoffman JI. Effect of right ventricular free wall ventriculotomy on right ventricular function: is that the correct question? J Thorac Cardiovasc Surg. (2014) 148(2):752–3. 10.1016/j.jtcvs.2014.04.002 25037944

[71] Heiberg J Ringgaard S Schmidt MR Redington A Hjortdal VE. Structural and functional alterations of the right ventricle are common in adults operated for ventricular septal defect as toddlers. Eur Heart J Cardiovasc Imaging. (2015) 16(5):483–9. 10.1093/ehjci/jeu292 25552465

[72] Kwok SY Yeung SS Li VW Cheung YF. Ventricular mechanics after repair of subarterial and perimembranous VSDs. Eur J Clin Invest. (2017) 47(12). 10.1111/eci.12852 29082523

[73] Friedberg MK Fernandes FP Roche SL Grosse-Wortmann L Manlhiot C Fackoury C Impaired right and left ventricular diastolic myocardial mechanics and filling in asymptomatic children and adolescents after repair of tetralogy of Fallot. Eur Heart J Cardiovasc Imaging. (2012) 13(11):905–13. 10.1093/ehjci/jes067 22467442

[74] Lurz P Puranik R Nordmeyer J Muthurangu V Hansen MS Schievano S Improvement in left ventricular filling properties after relief of right ventricle to pulmonary artery conduit obstruction: contribution of septal motion and interventricular mechanical delay. Eur Heart J. (2009) 30(18):2266–74. 10.1093/eurheartj/ehp258 19561027

[75] Egbe AC Adigun R Anand V West CP Montori VM Murad MH Left ventricular systolic dysfunction and cardiovascular outcomes in tetralogy of Fallot: systematic review and meta-analysis. Can J Cardiol. (2019) 35(12):1784–90. 10.1016/j.cjca.2019.07.634 31732195

[76] Broberg CS Aboulhosn J Mongeon FP Kay J Valente AM Khairy P Prevalence of left ventricular systolic dysfunction in adults with repaired tetralogy of fallot. Am J Cardiol. (2011) 107(8):1215–20. 10.1016/j.amjcard.2010.12.026 21349477

[77] Dubes V Benoist D Roubertie F Gilbert SH Constantin M Charron S Arrhythmogenic remodeling of the left ventricle in a porcine model of repaired tetralogy of Fallot. Circ Arrhythm Electrophysiol. (2018) 11(10):e006059. 10.1161/CIRCEP.117.006059 30354410 PMC6553519

[78] Janousek J Kovanda J Lozek M Tomek V Vojtovic P Gebauer R Pulmonary right ventricular resynchronization in congenital heart disease: acute improvement in right ventricular mechanics and contraction efficiency. Circ Cardiovasc Imaging. (2017) 10(9):e006424. 10.1161/CIRCIMAGING.117.006424 28877886

[79] Haggerty CM Suever JD Pulenthiran A Mejia-Spiegeler A Wehner GJ Jing L Association between left ventricular mechanics and diffuse myocardial fibrosis in patients with repaired tetralogy of Fallot: a cross-sectional study. J Cardiovasc Magn Reson. (2017) 19(1):100. 10.1186/s12968-017-0410-2 29228952 PMC5724335

[80] Jeong D Anagnostopoulos PV Roldan-Alzate A Srinivasan S Schiebler ML Wieben O Ventricular kinetic energy may provide a novel noninvasive way to assess ventricular performance in patients with repaired tetralogy of Fallot. J Thorac Cardiovasc Surg. (2015) 149(5):1339–47. 10.1016/j.jtcvs.2014.11.085 25623907 PMC4437857

[81] Fredriksson A Trzebiatowska-Krzynska A Dyverfeldt P Engvall J Ebbers T Carlhall CJ. Turbulent kinetic energy in the right ventricle: potential MR marker for risk stratification of adults with repaired tetralogy of Fallot. J Magn Reson Imaging. (2018) 47(4):1043–53. 10.1002/jmri.25830 28766919

[82] Jones M Ferrans V. Myocardial degeneration in congenital heart disease: comparision of morphologic findings in young and old patients with congenital heart disease associated with muscular obstruction to right ventricular outflow. Am J Cardiol. (1977) 39:1051–63. 10.1016/S0002-9149(77)80221-X 141201

[83] Chowdhury UK Sathia S Ray R Singh R Pradeep KK Venugopal P. Histopathology of the right ventricular outflow tract and its relationship to clinical outcomes and arrhythmias in patients with tetralogy of Fallot. J Thorac Cardiovasc Surg. (2006) 132(2):270–7. 10.1016/j.jtcvs.2006.04.001 16872949

[84] Chowdhury UK Jha A Ray R Kalaivani M Hasija S Kumari L Histopathology of the right ventricular outflow tract and the relation to hemodynamics in patients with repaired tetralogy of Fallot. J Thorac Cardiovasc Surg. (2019) 158(4):1173–83.e5. 10.1016/j.jtcvs.2019.03.118 31133352

[85] Pradegan N Vida VL Geva T Stellin G White MT Sanders SP Myocardial histopathology in late-repaired and unrepaired adults with tetralogy of Fallot. Cardiovasc Pathol. (2016) 25(3):225–31. 10.1016/j.carpath.2016.02.001 26938796

[86] Farah MC Castro CR Moreira Vde M Binotto MA Guerra VC Riso Ade A The impact of preexisting myocardial remodeling on ventricular function early after tetralogy of Fallot repair. J Am Soc Echocardiogr. (2010) 23(9):912–8. 10.1016/j.echo.2010.06.008 20650609

[87] Sharma HS Peters TH Moorhouse MJ van der Spek PJ Bogers AJ. DNA Microarray analysis for human congenital heart disease. Cell Biochem Biophys. (2006) 44(1):1–9. 10.1385/CBB:44:1:001 16456230

[88] Peters TH Sharma V Yilmaz E Mooi WJ Bogers AJ Sharma HS. DNA Microarray and quantitative analysis reveal enhanced myocardial VEGF expression with stunted angiogenesis in human tetralogy of Fallot. Cell Biochem Biophys. (2013) 67(2):305–16. 10.1007/s12013-013-9710-9 23897578

[89] Xie M Li Y Cheng TO Wang X Dong N Nie X The effect of right ventricular myocardial remodeling on ventricular function as assessed by two-dimensional speckle tracking echocardiography in patients with tetralogy of Fallot: a single center experience from China. Int J Cardiol. (2015) 178:300–7. 10.1016/j.ijcard.2014.10.027 25453412

[90] Farah MC Castro CR Moreira VM Riso Ade A Lopes AA Aiello VD. The myocardium in tetralogy of Fallot: a histological and morphometric study. Arq Bras Cardiol. (2009) 92(3):160–7. 3–71. 10.1590/S0066-782X2009000300002 19390702

[91] Garbern JC Lee RT. Heart regeneration: 20 years of progress and renewed optimism. Dev Cell. (2022) 57(4):424–39. 10.1016/j.devcel.2022.01.012 35231426 PMC8896288

[92] Bossers GPL Gunthel M van der Feen DE Hagdorn QAJ Koop AC van Duijvenboden K Neuregulin-1 enhances cell-cycle activity, delays cardiac fibrosis, and improves cardiac performance in rat pups with right ventricular pressure load. J Thorac Cardiovasc Surg. (2022) 164(6):e493–e510. 10.1016/j.jtcvs.2021.10.045. 34922752

[93] Mohammadi M M Abouissa A Azizah I Xie Y Cordero J Shirvani A Induction of cardiomyocyte proliferation and angiogenesis protects neonatal mice from pressure overload-associated maladaptation. JCI Insight. (2019) 5(16):e128336. 10.1172/jci.insight.128336 31335322 PMC6777810

[94] Adao R Mendes-Ferreira P Maia-Rocha C Santos-Ribeiro D Rodrigues PG Vidal-Meireles A Neuregulin-1 attenuates right ventricular diastolic stiffness in experimental pulmonary hypertension. Clin Exp Pharmacol Physiol. (2019) 46(3):255–65. 10.1111/1440-1681.13043 30339273

[95] Mendes-Ferreira P Maia-Rocha C Adao R Mendes MJ Santos-Ribeiro D Alves BS Neuregulin-1 improves right ventricular function and attenuates experimental pulmonary arterial hypertension. Cardiovasc Res. (2016) 109(1):44–54. 10.1093/cvr/cvv244 26503987

[96] Liu H Zhang CH Ammanamanchi N Suresh S Lewarchik C Rao K Control of cytokinesis by beta-adrenergic receptors indicates an approach for regulating cardiomyocyte endowment. Sci Transl Med. (2019) 11(513):eaaw6419. 10.1126/scitranslmed.aaw6419 31597755 PMC8132604

[97] de Carvalho A Bassaneze V Forni MF Keusseyan AA Kowaltowski AJ Krieger JE. Early postnatal cardiomyocyte proliferation requires high oxidative energy metabolism. Sci Rep. (2017) 7(1):15434. 10.1038/s41598-017-15656-3 29133820 PMC5684334

[98] Babu-Narayan SV Kilner PJ Li W Moon JC Goktekin O Davlouros PA Ventricular fibrosis suggested by cardiovascular magnetic resonance in adults with repaired tetralogy of fallot and its relationship to adverse markers of clinical outcome. Circulation. (2006) 113(3):405–13. 10.1161/CIRCULATIONAHA.105.548727 16432072

[99] Cochet H Iriart X Allain-Nicolai A Camaioni C Sridi S Nivet H Focal scar and diffuse myocardial fibrosis are independent imaging markers in repaired tetralogy of Fallot. Eur Heart J Cardiovasc Imaging. (2019) 20(9):990–1003. 10.1093/ehjci/jez068 30993335 PMC6704392

[100] Deanfield JE Ho SY Anderson RH McKenna WJ Allwork SP Hallidie-Smith KA. Late sudden death after repair of tetralogy of Fallot: a clinicopathologic study. Circulation. (1983) 67(3):626–31. 10.1161/01.CIR.67.3.626 6821905

[101] Schwartz SM Gordon D Mosca RS Bove EL Heidelberger KP Kulik TJ. Collagen content in normal, pressure, and pressure-volume overloaded developing human hearts. Am J Cardiol. (1996) 77(9):734–8. 10.1016/S0002-9149(97)89208-9 8651125

[102] Jeewa A Manickaraj AK Mertens L Manlhiot C Kinnear C Mondal T Genetic determinants of right-ventricular remodeling after tetralogy of Fallot repair. Pediatr Res. (2012) 72(4):407–13. 10.1038/pr.2012.95 22797143

[103] Yamamura K Yuen D Hickey EJ He X Chaturvedi RR Friedberg MK Right ventricular fibrosis is associated with cardiac remodelling after pulmonary valve replacement. Heart. (2019) 105(11):855–63. 10.1136/heartjnl-2018-313961 30514732

[104] Schraut WH Kampman K Lamberti JL Freeburger M Anagnostopoulos C Glagov S. Myocardial protection from permanent injury during aortic cross-clamping: effectiveness of pharmacological cardiac arrest combined with topical cardiac hypothermia. Ann Thorac Surg. (1981) 31(3):224–32. 10.1016/S0003-4975(10)60930-7 6971075

[105] Reddy S Zhao M Hu DQ Fajardo G Katznelson E Punn R Physiologic and molecular characterization of a murine model of right ventricular volume overload. Am J Physiol Heart Circ Physiol. (2013) 304(10):H1314–27. 10.1152/ajpheart.00776.2012 23504182 PMC3652064

[106] Oken DE Boucek RJ. Quantitation of collagen in human myocardium. Circ Res. (1957) 5(4):357–61. 10.1161/01.RES.5.4.357 13447177

[107] Crnkovic S Egemnazarov B Damico R Marsh LM Nagy BM Douschan P Disconnect between fibrotic response and right ventricular dysfunction. Am J Respir Crit Care Med. (2019) 199(12):1550–60. 10.1164/rccm.201809-1737OC 30557518 PMC6580669

[108] Akazawa Y Fujioka T Ide H Yazaki K Honjo O Sun M Impaired right and left ventricular function and relaxation induced by pulmonary regurgitation are not reversed by tardive antifibrosis treatment. Am J Physiol Heart Circ Physiol. (2021) 321(1):H38–51. 10.1152/ajpheart.00467.2020 34048283

[109] Andersen S Nielsen-Kudsk JE Vonk Noordegraaf A de Man FS. Right ventricular fibrosis. Circulation. (2019) 139(2):269–85. 10.1161/CIRCULATIONAHA.118.035326 30615500

[110] Sutendra G Dromparis P Paulin R Zervopoulos S Haromy A Nagendran J A metabolic remodeling in right ventricular hypertrophy is associated with decreased angiogenesis and a transition from a compensated to a decompensated state in pulmonary hypertension. J Mol Med (Berl. (2013) 91(11):1315–27. 10.1007/s00109-013-1059-4 23846254

[111] Bogaard HJ Natarajan R Henderson SC Long CS Kraskauskas D Smithson L Chronic pulmonary artery pressure elevation is insufficient to explain right heart failure. Circulation. (2009) 120(20):1951–60. 10.1161/CIRCULATIONAHA.109.883843 19884466

[112] Sano M Minamino T Toko H Miyauchi H Orimo M Qin Y p53-induced inhibition of hif-1 causes cardiac dysfunction during pressure overload. Nature. (2007) 446(7134):444–8. 10.1038/nature05602 17334357

[113] Ghorbel MT Cherif M Jenkins E Mokhtari A Kenny D Angelini GD Transcriptomic analysis of patients with tetralogy of Fallot reveals the effect of chronic hypoxia on myocardial gene expression. J Thorac Cardiovasc Surg. (2010) 140(2):337–45.e26. 10.1016/j.jtcvs.2009.12.055 20416888 PMC2951593

[114] Frump AL Bonnet S de Jesus Perez VA Lahm T. Emerging role of angiogenesis in adaptive and maladaptive right ventricular remodeling in pulmonary hypertension. Am J Physiol Lung Cell Mol Physiol. (2018) 314(3):L443–60. 10.1152/ajplung.00374.2017 29097426 PMC5900357

[115] Zhang HS Wu QY Xu M Zhou YX Shui CX. Mitogen-activated protein kinase signal pathways play an important role in right ventricular hypertrophy of tetralogy of Fallot. Chin Med J (Engl). (2012) 125(13):2243–9. 22882842

[116] Zhao Y Kang X Gao F Guzman A Lau RP Biniwale R Gene-environment regulatory circuits of right ventricular pathology in tetralogy of fallot. J Mol Med (Berl). (2019) 97(12):1711–22. 10.1007/s00109-019-01857-y 31834445 PMC7942233

[117] Kaynak B von Heydebreck A Mebus S Seelow D Hennig S Vogel J Genome-wide array analysis of normal and malformed human hearts. Circulation. (2003) 107(19):2467–74. 10.1161/01.CIR.0000066694.21510.E2 12742993

[118] Szklarczyk D Gable AL Lyon D Junge A Wyder S Huerta-Cepas J STRING V11: protein-protein association networks with increased coverage, supporting functional discovery in genome-wide experimental datasets. Nucleic Acids Res. (2019) 47(D1):D607–13. 10.1093/nar/gky1131 30476243 PMC6323986

[119] Adamo L Rocha-Resende C Prabhu SD Mann DL. Reappraising the role of inflammation in heart failure. Nat Rev Cardiol. (2020) 17(5):269–85. 10.1038/s41569-019-0315-x 31969688

[120] Konstantinov IE Coles JG Boscarino C Takahashi M Goncalves J Ritter J Gene expression profiles in children undergoing cardiac surgery for right heart obstructive lesions. J Thorac Cardiovasc Surg. (2004) 127(3):746–54. 10.1016/j.jtcvs.2003.08.056 15001903

[121] Xia Y Hong H Ye L Wang Y Chen H Liu J. Label-free quantitative proteomic analysis of right ventricular remodeling in infant tetralogy of Fallot patients. J Proteomics. (2013) 84:78–91. 10.1016/j.jprot.2013.03.032 23571024

[122] Charron S Roubertie F Benoist D Dubes V Gilbert SH Constantin M Identification of region-specific myocardial gene expression patterns in a chronic swine model of repaired tetralogy of Fallot. PLoS One. (2015) 10(8):e0134146. 10.1371/journal.pone.0134146 26252659 PMC4529093

[123] Teerlink JR Diaz R Felker GM McMurray JJV Metra M Solomon SD Cardiac myosin activation with omecamtiv mecarbil in systolic heart failure. N Engl J Med. (2021) 384(2):105–16. 10.1056/NEJMoa2025797 33185990

[124] Lookin O Kuznetsov D Protsenko Y. Omecamtiv mecarbil attenuates length-tension relationship in healthy rat myocardium and preserves it in monocrotaline-induced pulmonary heart failure. Clin Exp Pharmacol Physiol. (2022) 49(1):84–93. 10.1111/1440-1681.13584 34459025

[125] Biering-Sorensen T Minamisawa M Liu J Claggett B Papolos AI Felker GM The effect of the cardiac myosin activator, omecamtiv mecarbil, on right ventricular structure and function in chronic systolic heart failure (COSMIC-HF). Eur J Heart Fail. (2021) 23(6):1052–6. 10.1002/ejhf.2181 33826209

[126] Oumeiri B E van de Borne P Hubesch G Herpain A Annoni F Jespers P The myosin activator omecamtiv mecarbil improves wall stress in a rat model of chronic aortic regurgitation. Physiol Rep. (2021) 9(16):e14988. 10.14814/phy2.14988 34405966 PMC8371349

[127] Bakkehaug JP Kildal AB Engstad ET Boardman N Naesheim T Ronning L Myosin activator omecamtiv mecarbil increases myocardial oxygen consumption and impairs cardiac efficiency mediated by resting myosin ATPase activity. Circ Heart Fail. (2015) 8(4):766–75. 10.1161/CIRCHEARTFAILURE.114.002152 26025342

[128] Merkus D Kajiya F Vink H Vergroesen I Dankelman J Goto M Prolonged diastolic time fraction protects myocardial perfusion when coronary blood flow is reduced. Circulation. (1999) 100(1):75–81. 10.1161/01.CIR.100.1.75 10393684

[129] Doty DB Wright CB Hiratzka LF Eastham CL Marcus ML. Coronary reserve in volume-induced right ventricular hypertrophy from atrial septal defect. Am J Cardiol. (1984) 54(8):1059–63. 10.1016/S0002-9149(84)80144-7 6238518

[130] Teerlink JR Malik FI Kass DA. Letter by teerlink, et al regarding article, “myosin activator omecamtiv mecarbil increases myocardial oxygen consumption and impairs cardiac efficiency mediated by resting myosin ATPase activity”. Circ Heart Fail. (2015) 8(6):1141. 10.1161/CIRCHEARTFAILURE.115.002492 26578671 PMC4654674

[131] Iacobazzi D Suleiman MS Ghorbel M George SJ Caputo M Tulloh RM. Cellular and molecular basis of RV hypertrophy in congenital heart disease. Heart. (2016) 102(1):12–7. 10.1136/heartjnl-2015-308348 26516182 PMC4717403

[132] Koop AC Bossers GPL Ploegstra MJ Hagdorn QAJ Berger RMF Sillje HHW Metabolic remodeling in the pressure-loaded right ventricle: shifts in glucose and fatty acid metabolism-A systematic review and meta-analysis. J Am Heart Assoc. (2019) 8(21):e012086. 10.1161/JAHA.119.012086 31657265 PMC6898858

[133] Nagendran J Gurtu V Fu DZ Dyck JR Haromy A Ross DB A dynamic and chamber-specific mitochondrial remodeling in right ventricular hypertrophy can be therapeutically targeted. J Thorac Cardiovasc Surg. (2008) 136(1):168–78. 78.e1–3. 10.1016/j.jtcvs.2008.01.040 18603070

[134] Piao L Fang YH Cadete VJ Wietholt C Urboniene D Toth PT The inhibition of pyruvate dehydrogenase kinase improves impaired cardiac function and electrical remodeling in two models of right ventricular hypertrophy: resuscitating the hibernating right ventricle. J Mol Med (Berl). (2010) 88(1):47–60. 10.1007/s00109-009-0524-6 19949938 PMC3155251

[135] Sun XQ Zhang R Zhang HD Yuan P Wang XJ Zhao QH Reversal of right ventricular remodeling by dichloroacetate is related to inhibition of mitochondria-dependent apoptosis. Hypertens Res. (2016) 39(5):302–11. 10.1038/hr.2015.153 26763846

[136] Spyropoulos F Michael Z Finander B Vitali S Kosmas K Zymaris P Acetazolamide Improves right ventricular function and metabolic gene dysregulation in experimental pulmonary arterial hypertension. Front Cardiovasc Med. (2021) 8:662870. 10.3389/fcvm.2021.662870 34222363 PMC8247952

[137] Fang YH Piao L Hong Z Toth PT Marsboom G Bache-Wiig P Therapeutic inhibition of fatty acid oxidation in right ventricular hypertrophy: exploiting randle's cycle. J Mol Med (Berl). (2012) 90(1):31–43. 10.1007/s00109-011-0804-9 21874543 PMC3249482

[138] Kan Z Yan W Wang N Fang Y Gao H Song Y. Identification of circRNA-miRNA-mRNA regulatory network and crucial signaling pathway axis involved in tetralogy of Fallot. Front Genet. (2022) 13:917454. 10.3389/fgene.2022.917454 35873466 PMC9300927

[139] Abu-Halima M Meese E Keller A Abdul-Khaliq H Radle-Hurst T. Analysis of circulating microRNAs in patients with repaired tetralogy of Fallot with and without heart failure. J Transl Med. (2017) 15(1):156. 10.1186/s12967-017-1255-z 28693530 PMC5504636

[140] Liang D Xu X Deng F Feng J Zhang H Liu Y miRNA-940 reduction contributes to human tetralogy of Fallot development. J Cell Mol Med. (2014) 18(9):1830–9. 10.1111/jcmm.12309 24889693 PMC4196658

[141] O'Brien JE Jr. Kibiryeva N Zhou XG Marshall JA Lofland GK Artman M Noncoding RNA expression in myocardium from infants with tetralogy of Fallot. Circ Cardiovasc Genet. (2012) 5(3):279–86. 10.1161/CIRCGENETICS.111.961474 22528145

[142] Zhang J Chang JJ Xu F Ma XJ Wu Y Li WC MicroRNA deregulation in right ventricular outflow tract myocardium in nonsyndromic tetralogy of fallot. Can J Cardiol. (2013) 29(12):1695–703. 10.1016/j.cjca.2013.07.002 24140236

[143] Zhou H Lin S Li X Guo D Wang Y Hu Y. Serum miR-222 is independently associated with atrial fibrillation in patients with degenerative valvular heart disease. BMC Cardiovasc Disord. (2021) 21(1):98. 10.1186/s12872-021-01909-7 33593281 PMC7885218

[144] Knyrim M Rabe S Grossmann C Gekle M Schreier B. Influence of miR-221/222 on cardiomyocyte calcium handling and function. Cell Biosci. (2021) 11(1):160. 10.1186/s13578-021-00676-4 34404451 PMC8369661

[145] Li C Zhao J Sun W. microRNA-222-Mediated VHL downregulation facilitates retinoblastoma chemoresistance by increasing HIF1alpha expression. Invest Ophthalmol Vis Sci. (2020) 61(10):9. 10.1167/iovs.61.10.9 32756923 PMC7441340

[146] Liu X Xiao J Zhu H Wei X Platt C Damilano F miR-222 is necessary for exercise-induced cardiac growth and protects against pathological cardiac remodeling. Cell Metab. (2015) 21(4):584–95. 10.1016/j.cmet.2015.02.014 25863248 PMC4393846

[147] Chen L Han Y Li Y Chen B Bai X Belguise K Hepatocyte-derived exosomal MiR-194 activates PMVECs and promotes angiogenesis in hepatopulmonary syndrome. Cell Death Dis. (2019) 10(11):853. 10.1038/s41419-019-2087-y 31700002 PMC6838168

[148] Reddy S Hu DQ Zhao M Blay E Jr. Sandeep N Ong SG miR-21 is associated with fibrosis and right ventricular failure. JCI Insight. (2017) 2(9):e91625. 10.1172/jci.insight.91625 28469078 PMC5414555

[149] van den Bosch E van Genuchten WJ Luijnenburg SE Duppen N Kamphuis VP Roos-Hesselink JW Associations between blood biomarkers, cardiac function and adverse outcome in a young tetralogy of Fallot cohort. Int J Cardiol. (2022) 361:31–7. 10.1016/j.ijcard.2022.04.065 35487320

[150] Konstam MA Kiernan MS Bernstein D Bozkurt B Jacob M Kapur NK Evaluation and management of right-sided heart failure: a scientific statement from the American heart association. Circulation. (2018) 137(20):e578–622. 10.1161/CIR.0000000000000560 29650544

[151] Budts W Roos-Hesselink J Radle-Hurst T Eicken A McDonagh TA Lambrinou E Treatment of heart failure in adult congenital heart disease: a position paper of the working group of grown-up congenital heart disease and the heart failure association of the European society of cardiology. Eur Heart J. (2016) 37(18):1419–27. 10.1093/eurheartj/ehv741 26787434 PMC4914888

[152] Peng H Carretero OA Vuljaj N Liao TD Motivala A Peterson EL Angiotensin-converting enzyme inhibitors: a new mechanism of action. Circulation. (2005) 112(16):2436–45. 10.1161/CIRCULATIONAHA.104.528695 16216963 PMC6824430

[153] Babu-Narayan SV Uebing A Davlouros PA Kemp M Davidson S Dimopoulos K Randomised trial of ramipril in repaired tetralogy of Fallot and pulmonary regurgitation: the APPROPRIATE study (ace inhibitors for potential PRevention of the deleterious effects of pulmonary regurgitation in adults with repaired TEtralogy of Fallot). Int J Cardiol. (2012) 154(3):299–305. 10.1016/j.ijcard.2010.09.057 20970202

[154] Maron BA Leopold JA. The role of the renin-angiotensin-aldosterone system in the pathobiology of pulmonary arterial hypertension (2013 grover conference series). Pulm Circ. (2014) 4(2):200–10. 10.1086/675984 25006439 PMC4070776

[155] Friedberg MK Cho MY Li J Assad RS Sun M Rohailla S Adverse biventricular remodeling in isolated right ventricular hypertension is mediated by increased transforming growth factor-beta1 signaling and is abrogated by angiotensin receptor blockade. Am J Respir Cell Mol Biol. (2013) 49(6):1019–28. 10.1165/rcmb.2013-0149OC 23841477

[156] Sharifi Kia D Benza E Bachman TN Tushak C Kim K Simon MA. Angiotensin receptor-neprilysin inhibition attenuates right ventricular remodeling in pulmonary hypertension. J Am Heart Assoc. (2020) 9(13):e015708. 10.1161/JAHA.119.015708 32552157 PMC7670537

[157] Appadurai V Thoreau J Malpas T Nicolae M. Sacubitril/valsartan in adult congenital heart disease patients with chronic heart failure - A single centre case series and call for an international registry. Heart Lung Circ. (2020) 29(1):137–41. 10.1016/j.hlc.2018.12.003 30686641

[158] Zandstra TE Nederend M Jongbloed MRM Kies P Vliegen HW Bouma BJ Sacubitril/valsartan in the treatment of systemic right ventricular failure. Heart. (2021) 107(21):1725–30. 10.1136/heartjnl-2020-318074 33452121 PMC8522462

[159] Cardoso R Graffunder FP Ternes CMP Fernandes A Rocha AV Fernandes G SGLT2 Inhibitors decrease cardiovascular death and heart failure hospitalizations in patients with heart failure: a systematic review and meta-analysis. EClinicalMedicine. (2021) 36:100933. 10.1016/j.eclinm.2021.100933 34308311 PMC8257984

[160] Chowdhury B Luu AZ Luu VZ Kabir MG Pan Y Teoh H The SGLT2 inhibitor empagliflozin reduces mortality and prevents progression in experimental pulmonary hypertension. Biochem Biophys Res Commun. (2020) 524(1):50–6. 10.1016/j.bbrc.2020.01.015 31980166

[161] Tang Y Tan S Li M Tang Y Xu X Zhang Q Dapagliflozin, sildenafil and their combination in monocrotaline-induced pulmonary arterial hypertension. BMC Pulm Med. (2022) 22(1):142. 10.1186/s12890-022-01939-7 35413880 PMC9006601

[162] Li H Zhang Y Wang S Yue Y Liu Q Huang S Dapagliflozin has No protective effect on experimental pulmonary arterial hypertension and pulmonary trunk banding rat models. Front Pharmacol. (2021) 12:756226. 10.3389/fphar.2021.756226 34790128 PMC8591217

[163] Egorova AD Nederend M Tops LF Vliegen HW Jongbloed MRM Kies P. The first experience with sodium-glucose cotransporter 2 inhibitor for the treatment of systemic right ventricular failure. ESC Heart Fail. (2022) 9(3):2007–12. 10.1002/ehf2.13871 35355435 PMC9065858

[164] Borgdorff MA Bartelds B Dickinson MG Boersma B Weij M Zandvoort A Sildenafil enhances systolic adaptation, but does not prevent diastolic dysfunction, in the pressure-loaded right ventricle. Eur J Heart Fail. (2012) 14(9):1067–74. 10.1093/eurjhf/hfs094 22730335

[165] Nagendran J Archer SL Soliman D Gurtu V Moudgil R Haromy A Phosphodiesterase type 5 is highly expressed in the hypertrophied human right ventricle, and acute inhibition of phosphodiesterase type 5 improves contractility. Circulation. (2007) 116(3):238–48. 10.1161/CIRCULATIONAHA.106.655266 17606845

[166] Borgdorff MA Bartelds B Dickinson MG van Wiechen MP Steendijk P de Vroomen M Sildenafil treatment in established right ventricular dysfunction improves diastolic function and attenuates interstitial fibrosis independent from afterload. Am J Physiol Heart Circ Physiol. (2014) 307(3):H361–9. 10.1152/ajpheart.00843.2013 24878769

[167] Bishu K Hamdani N Mohammed SF Kruger M Ohtani T Ogut O Sildenafil and B-type natriuretic peptide acutely phosphorylate titin and improve diastolic distensibility in vivo. Circulation. (2011) 124(25):2882–91. 10.1161/CIRCULATIONAHA.111.048520 22144574 PMC3412357

[168] Janousek J Kovanda J Lozek M Tomek V Gebauer R Kubus P Cardiac resynchronization therapy for treatment of chronic subpulmonary right ventricular dysfunction in congenital heart disease. Circ Arrhythm Electrophysiol. (2019) 12(5):e007157. 10.1161/CIRCEP.119.007157 30991822

[169] Geva T Gauvreau K Powell AJ Cecchin F Rhodes J Geva J Randomized trial of pulmonary valve replacement with and without right ventricular remodeling surgery. Circulation. (2010) 122(11 Suppl):S201–8. 10.1161/CIRCULATIONAHA.110.951178 20837914 PMC2943672

[170] Blum KM Mirhaidari GJM Breuer CK. Tissue engineering: relevance to neonatal congenital heart disease. Semin Fetal Neonatal Med. (2022) 27(1):101225. 10.1016/j.siny.2021.101225 33674254 PMC8390581

[171] Yu H Del Nido PJ Geva T Yang C Wu Z Rathod RH Multi-Band surgery for repaired tetralogy of Fallot patients with reduced right ventricle ejection fraction: a pilot study. Front Physiol. (2020) 11:198. 10.3389/fphys.2020.00198 32265727 PMC7103653

[172] Loomba RS Buelow MW Woods RK. Complete repair of tetralogy of Fallot in the neonatal versus non-neonatal period: a meta-analysis. Pediatr Cardiol. (2017) 38(5):893–901. 10.1007/s00246-017-1579-8 28190140

[173] Fleg JL Cooper LS Borlaug BA Haykowsky MJ Kraus WE Levine BD Exercise training as therapy for heart failure: current status and future directions. Circ Heart Fail. (2015) 8(1):209–20. 10.1161/CIRCHEARTFAILURE.113.001420 25605639 PMC4802377

[174] Hieda M Sarma S Hearon CM Jr. MacNamara JP Dias KA Samels M One-Year committed exercise training reverses abnormal left ventricular myocardial stiffness in patients with stage B heart failure with preserved ejection fraction. Circulation. (2021) 144(12):934–46. 10.1161/CIRCULATIONAHA.121.054117 34543068 PMC8849598

[175] Cai MX Shi XC Chen T Tan ZN Lin QQ Du SJ Exercise training activates neuregulin 1/ErbB signaling and promotes cardiac repair in a rat myocardial infarction model. Life Sci. (2016) 149:1–9. 10.1016/j.lfs.2016.02.055 26892146

[176] Vujic A Lerchenmuller C Wu TD Guillermier C Rabolli CP Gonzalez E Exercise induces new cardiomyocyte generation in the adult mammalian heart. Nat Commun. (2018) 9(1):1659. 10.1038/s41467-018-04083-1 29695718 PMC5916892

[177] Duppen N Etnel JR Spaans L Takken T van den Berg-Emons RJ Boersma E Does exercise training improve cardiopulmonary fitness and daily physical activity in children and young adults with corrected tetralogy of Fallot or fontan circulation? A randomized controlled trial. Am Heart J. (2015) 170(3):606–14. 10.1016/j.ahj.2015.06.018 26385046

[178] Duppen N Kapusta L de Rijke YB Snoeren M Kuipers IM Koopman LP The effect of exercise training on cardiac remodelling in children and young adults with corrected tetralogy of Fallot or fontan circulation: a randomized controlled trial. Int J Cardiol. (2015) 179:97–104. 10.1016/j.ijcard.2014.10.031 25464424

[179] Duppen N Takken T Hopman MT ten Harkel AD Dulfer K Utens EM Systematic review of the effects of physical exercise training programmes in children and young adults with congenital heart disease. Int J Cardiol. (2013) 168(3):1779–87. 10.1016/j.ijcard.2013.05.086 23746621

[180] Scheffers LE Berg L Ismailova G Dulfer K Takkenberg JJM Helbing WA. Physical exercise training in patients with a fontan circulation: a systematic review. Eur J Prev Cardiol. (2021) 28(11):1269–78. 10.1177/2047487320942869 34551076

[181] Novakovic M Prokselj K Rajkovic U Vizintin Cuderman T Jansa Trontelj K Fras Z Exercise training in adults with repaired tetralogy of Fallot: a randomized controlled pilot study of continuous versus interval training. Int J Cardiol. (2018) 255:37–44. 10.1016/j.ijcard.2017.12.105 29338917

[182] Duppen N Geerdink LM Kuipers IM Bossers SS Koopman LP van Dijk AP Regional ventricular performance and exercise training in children and young adults after repair of tetralogy of Fallot: randomized controlled pilot study. Circ Cardiovasc Imaging. (2015) 8(4):e002006. 10.1161/CIRCIMAGING.114.002006 25784723

