# Supplementary material for: The right ventricle in tetralogy of Fallot: adaptation to sequential loading
Source: Front Pediatr. 2023 Mar 16;11:1098248. doi: 10.3389/fped.2023.1098248 (PMC10061113; doi:10.3389/fped.2023.1098248)
Supplement: Supplementary file 1 [file Datasheet1.pdf]

Supplementary Material

1 Supplementary Figures and Tables

1.1 Supplementary Figures

Supplementary Figure 1. Clusters of differentially expressed genes in right ventricular myocardium from studies comparing Tetralogy of Fallot patients to controls (21, 48-50).

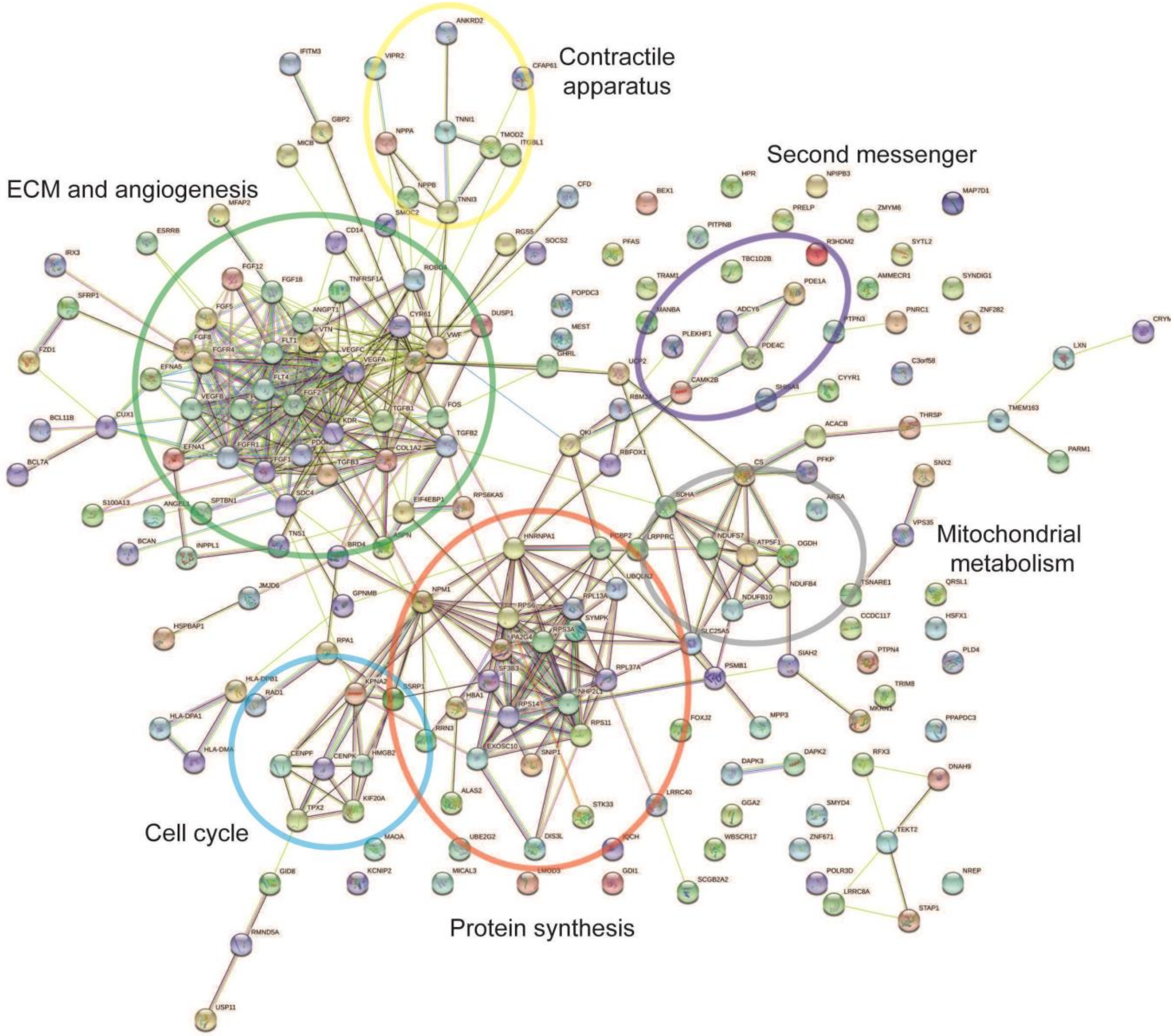

1.2 Supplementary Tables

Supplementary Table 2. Lists of differentially expressed genes in right ventricular myocardium from studies comparing Tetralogy of Fallot patients to controls (21, 48-50).

Zhao et al. (2019)(49)	Peters et al. (2013)(21)	Zhang et al. (2012)(48)	Kaynak et al. (2003)(50)
3-Mar	VEGFA	ALAS2	A2BP1
BEX1	VEGFB	ANKRD2	ACACB
BRD4	VEGFC	ASPN	ADCY6
CCDC117	VEGFD	C20orf26	AMMECR1
DAPK3	FLT1	C5orf13	ARSA
EFNA5	KDR	CD14	ATP5F1
ESRRB	FLT4	CENPF	BCL11B
FGF18	ANGPT1	COL1A2	BCL7A
FOXJ2	TNF	CRYM	BRDG1
FZD1	TNFR1	CYR61	CAMK2B
GGA2	TGFB1	DAPK2	CS
GID8	TGFB2	DF	CYYR1
GPNMB	TGFB2R	DUSP1	D1346
GPNMB	TGFB3	EIF4EBP1	DIA1
HMGB2	TGFB3R	FOS	DKFZP566
HNRNPA1	PDGFA	GBP2	DNAH9
IRX3	EFNA1	GHRL_HUMAN	FKSG14
ITGBL1	FGF1	HBA1	FLJ10350
MANBA	FGF2	HLA-DMA	FLJ10989
MKRN1	FGF5	HLA-DPA1	FLJ20312
NPM1	FGF8	HLA-DPB1	FLJ20331
PARM1	FGF8	HPR	FLJ22623
PFKP	FGF12	KCNIP2	GDI1
PITPNB	FGF18	KIF20A	IFITM3
PLD4	FGFR1	KPNA2	INPPL1
PNRC1	FGFR4	LMOD3	KIAA0220
POLR3D		MAOA	KIAA0759

POPDC3		MEST	KIAA1055
R3HDM2		MFAP2	KIAA1353
RMND5A		MPP3	KIAA1437
RPA1		NP_073621	LOC51177
SDC4		NP_112185	LOC51189
SF3B3		NP_659440	LOC51244
SFRP1		NPPA	LRPPRC
SHISA4		NPPB	LW-1
SIAH2		O94909	LXN
SLC25A5		PIPI_HUMAN	MGC10600
SMYD4		PLEKHF1	MGC12921
SOCS2		PRELP	MGC13038
SSRP1		PTDSR	MGC2664
SYMPK		PTPN3	MGC4562
SYNDIG1		Q8TCB4	MICB
TNNI1		Q9NT46	MSTP032
UCP2		RBM24	NDUFB10
USP11		SMOC2	NDUFB4
VIPR2		SPTBN1	NDUFS7
WBSCR17		TNS	NHP2L1
ZNF282		TPX2	OGDH
ZNF671		VTN	PA2G4
			PCBP2
			PDE1A
			PDE4C
			PFAS
			PMSCL2
			PSMB1
			PTPN4
			QKI
			RAD1
			RFX3
			RNF27

			ROBO4
			RPL13A
			RPL37A
			RPS11
			RPS14
			RPS3A
			RPS6
			RPS6KA5
			RRN3
			S100A13
			SCGB2A2
			SDHA
			SNIP1
			SNX2
			STK33
			SYTL2
			TEKT2
			THRSP
			TMOD2
			TNNI1
			TNNI3
			TRAM
			UBE2G2
			UBQLN2
			VPS35
			VWF
